# The ETS transcription factor ELF1 regulates a broadly antiviral program distinct from the type I interferon response

**DOI:** 10.1371/journal.ppat.1007634

**Published:** 2019-11-04

**Authors:** Leon Louis Seifert, Clara Si, Debjani Saha, Mohammad Sadic, Maren de Vries, Sarah Ballentine, Aaron Briley, Guojun Wang, Ana M. Valero-Jimenez, Adil Mohamed, Uwe Schaefer, Hong M. Moulton, Adolfo García-Sastre, Shashank Tripathi, Brad R. Rosenberg, Meike Dittmann

**Affiliations:** 1 Laboratory of Virology and Infectious Disease, The Rockefeller University, New York, New York, United States of America; 2 Department of Microbiology, New York University School of Medicine, New York, New York, United States of America; 3 Department of Microbiology, Icahn School of Medicine at Mount Sinai, New York, New York, United States of America; 4 Global Health and Emerging Pathogens Institute, Icahn School of Medicine at Mount Sinai, New York, New York, United States of America; 5 Laboratory of Immune Cell Epigenetics and Signaling, The Rockefeller University, New York, New York, United States of America; 6 Carlson College of Veterinary Medicine, Oregon State University, Corvallis, Oregon, United States of America; 7 Department of Medicine, Division of Infectious Diseases, Icahn School of Medicine at Mount Sinai, New York, New York, United States of America; 8 The Tisch Cancer Institute, Icahn School of Medicine at Mount Sinai, New York, New York, United States of America; 9 Microbiology and Cell Biology Department, Centre for Infectious Disease Research, Indian Institute of Science, Bangalore, India; Institute for Virus Research, Kyoto University, JAPAN

## Abstract

Induction of vast transcriptional programs is a central event of innate host responses to viral infections. Here we report a transcriptional program with potent antiviral activity, driven by E74-like ETS transcription factor 1 (ELF1). Using microscopy to quantify viral infection over time, we found that ELF1 inhibits eight diverse RNA and DNA viruses after multi-cycle replication. Elf1 deficiency results in enhanced susceptibility to influenza A virus infections in mice. ELF1 does not feed-forward to induce interferons, and ELF1’s antiviral effect is not abolished by the absence of STAT1 or by inhibition of JAK phosphorylation. Accordingly, comparative expression analyses by RNA-seq revealed that the ELF1 transcriptional program is distinct from interferon signatures. Thus, ELF1 provides an additional layer of the innate host response, independent from the action of type I interferons.

## Author summary

After decades of research on the innate immune system, we still struggle to understand exactly how this first line of defense protects cells against viral infections. Our gap in knowledge stems, on one hand, from the sheer number of effector genes, few of which have been characterized in mechanistic detail. On the other hand, our understanding of innate gene transcription is constantly evolving. We know that different regulatory mechanisms greatly influence the quality, magnitude, and timing of gene expression, all of which may contribute to the antiviral power of the innate response. Deciphering these regulatory mechanisms is indispensable for harnessing the power of innate immunity in novel antiviral therapies. Here, we report a novel transcriptional program as part of the cell-intrinsic immune system, raised by E74-like ETS transcription factor 1 (ELF1). ELF1 potently restricts multi-cycle propagation of all viruses tested in our study. Reduced levels of ELF1 significantly diminish host defenses against influenza A virus *in vitro* and *in vivo*, suggesting a critical but previously overlooked role of this ETS transcription factor. The ELF1 program is complex and comprises over 300 potentially antiviral genes, which are almost entirely distinct from those known to be induced by interferon. Taken together, our data provide evidence for a program of antiviral protection that expands the previously known arsenal of the innate immune response.

## Introduction

The innate immune system provides a first line of defense against viral infections, achieving its remarkable potency through the establishment of complex transcriptional programs. Some of the best-studied innate transcriptional programs are those brought upon by immune messenger proteins called type I interferons, and comprise hundreds of genes (interferon-stimulated genes, ISGs) [1]. The few ISGs that have been mechanistically characterized achieve their antiviral power by acting on different stages of viral life cycles, from entry to viral genome replication, assembly, egress and finally, maturation [1,2]. Given the sheer number of ISGs and their mechanistic diversity, we are still striving to understand the complex means by which ISGs confer broad protection against enveloped and non-enveloped RNA and DNA viruses, and even intracellular bacteria and parasites [3–5]. In-depth understanding of distinct transcriptional programs and their specific contributions to innate immune barriers are crucial for defining the nature of host defenses.

The predominant transcription factors involved in type I interferon production and signaling are signal transducers and activators of transcription (*STAT*s) and interferon-regulatory factors (*IRF*s). Triggered by pathogen sensing and pattern recognition pathways, *IRF3* and *IRF7* bind to interferon promoters to initiate interferon production [6]. Upon interferon signal transduction, phosphorylated STAT1/2 and IRF9 form the interferon-stimulated gene factor 3 (ISGF3) complex, which localizes to interferon-sensitive response elements (ISREs) and initiates the transcription of ISGs [7,8]. In addition to IRF3, IRF7, and IRF9, another notable IRF within the innate immune response is IRF1. IRF1, itself an ISG, has broad and potent antiviral activity against a wide variety of RNA and DNA viruses [3,4]. Remarkably, this activity appears to be mediated through direct transcriptional initiation of a subset of ISGs, even in the absence of interferon or intact interferon signaling [4]. The aforementioned transcription factors are well-established as critical regulators, and, with the exception of IRF9, have been validated by individual knockouts resulting in enhanced viral susceptibility *in vivo* [9–12]. Notably, IRF7 was the first innate transcription factor functionally associated with enhanced susceptibility to influenza virus infections in patients [13].

Primarily, members of the ETS transcription factor family have been linked to diverse roles in lymphocyte development, cancer, and angiogenesis, and less so as regulators in cell-intrinsic antiviral pathways [14–17]. However, one comprehensive study characterized E74-like ETS transcription factor 4 (*ELF4*) as a broadly antiviral regulator *in vitro* and *in vivo* [18]. It was shown to be a component of the IRF3/7 pathway, and bound the interferon-beta promoter to enhance interferon-beta expression. This feed-forward mechanism to produce interferon is ELF4’s proposed mechanism of antiviral action. Other ELFs tested in that study (ELF1, 2, 3 and 5) did not enhance expression of interferon, suggesting that different ELF proteins have distinct functions.

Here, we characterize another member of the ETS transcription factor family as a cell-intrinsic element of the antiviral immune response. We show that ELF1 regulates expression of a previously unidentified set of genes, which results in a potent antiviral state both *in vitro* and *in vivo*. Unlike ELF4, ELF1 retains its broad antiviral activity in the absence of interferon or interferon signaling, and the pool of ELF1 differentially expressed genes minimally overlaps with those regulated by interferon. Overall, our data suggest that ELF1 controls a novel and unique innate immune program.

## Results

### ELF1 mRNA is upregulated by type I interferon and upon viral infection

Multiple signal transduction pathways, triggered by external stimuli, relay their signals to members of the ETS transcription factor family [16]. For family member ELF1, one known trigger is type I interferon, as liver biopsies of patients receiving interferon-alpha showed upregulated ELF1 mRNA [19]. Subsequently, ELF1 was previously used as part of a cDNA library assembled to screen for antiviral function of individual ISGs [4]. Using that library, we were able to demonstrate for the first time that ELF1, in epithelial cells, can inhibit a virus [2].

**Fig 1:**
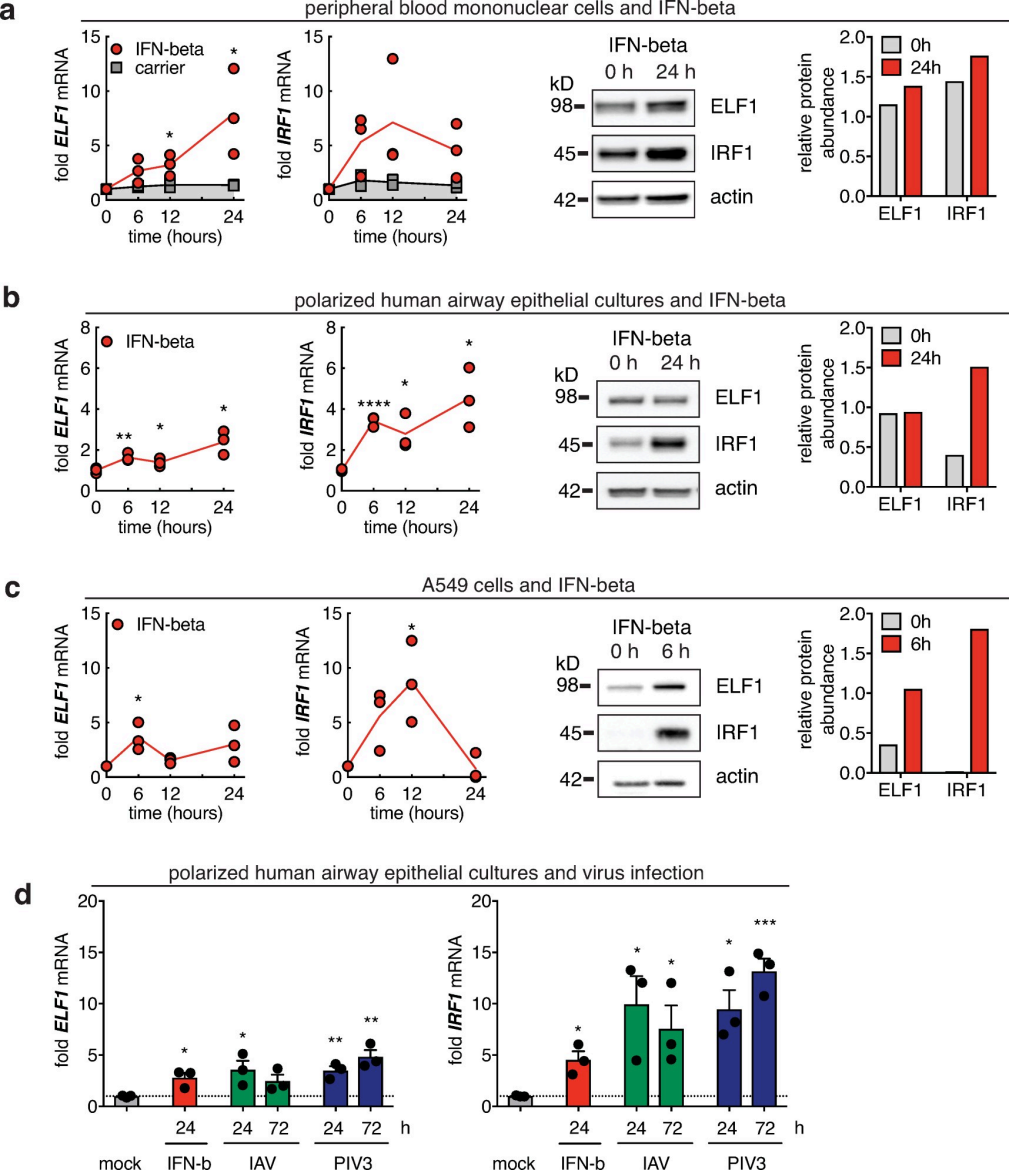
Intrinsic and innate expression of transcription factor ELF1. **a.–c.** Cells were treated with 500 U/ml IFN–beta, and mRNA (left side) or protein (right side) of ELF1 or control ISG IRF1 measured over time. mRNA was determined by RT–qPCR and normalized relative to housekeeping gene RPS–11. Fold increase over pre–treatment control levels from n = 3 independent replicates; data shown as individual replicates; line represents the mean. Paired t–test of each time point compared to control, *p<0.1, **p<0.01, ***p<0.001. Western blots showing ELF1 or IRF1 at ELF1 peak mRNA time point; protein levels quantified by densitometry, normalized to actin control. **a.** Peripheral blood mononuclear cells (PBMC) from n = 3 donors, BSA as carrier control; **b.** human airway epithelial cultures (basolaterally stimulated); **c.** A549 cells. **d.** Human airway epithelial cultures were treated basolaterally with 500 U/ml of IFN–beta or infected apically with influenza A virus (IAV) or parainfluenzavirus 3 (PIV3). mRNA of ELF1 and IRF1 determined at 24 or / and 72 h post treatment or infection as described in (**a**). RT–qPCR data shown as mean of n = 3 biological replicates.

To establish ELF1 as a component of the antiviral immune response, we determined its expression in different cell lines, primary cell culture systems, and a mouse model, upon interferon treatment, stimulation with pathogen-associated molecular patterns (PAMPs), or viral infection (Fig 1 and S1 Fig).

We first challenged different in vitro models with interferon-beta, and then measured mRNA levels of ELF1 or IRF1, an interferon-stimulated transcription factor [20], over time (Fig 1A–1C, mRNA levels shown in left side panels). On the mRNA level, both IRF1 and ELF1 mRNA were upregulated in response to interferon in peripheral blood mononuclear cells (PBMC), in stratified human airway epithelium cultures—which closely mimic the human airway epithelium in vivo in terms of cell types, architecture, and polarization [21]—and in A549 lung carcinoma epithelial cells (Fig 1A–1C). Our results confirm that like other ISGs, ELF1 mRNA is significantly upregulated post-interferon treatment. Interestingly, in A549 cells, ELF1 and IRF1 expression peaked at 6 h post interferon treatment, which was earlier than observed in PBMC and airway epithelial cultures. The difference in timing might reflect the heterogeneous response of different cell types represented in PBMC and airway cultures, as opposed to the homogenous response in the clonal cell line A549.

Next, we determined whether the observed mRNA upregulation correlated with levels of ELF1 protein. We performed western blot analyses probing for ELF1 or IRF1 at steady-state or upon interferon-stimulation, at the time point of maximum ELF1 mRNA upregulation (Fig 1A–1C, protein levels shown in right side panels, S1A–S1C Fig). Our first observation was that ELF1 protein was readily detectable at steady state in all cells or cell systems tested (PBMC, airway epithelium cultures, A549, primary normal human bronchioepithelial (NHBE) cells, 293T, and HeLa cells) (Fig 1A–1C, S1A–S1C Fig). IRF1 protein was also detectable at steady-state, albeit only in PBMC, airway epithelium cultures, and, at very low abundance, A549 (Fig 1A–1C, S1A–S1C Fig). Our second observation was that ELF1 protein levels did not always correlate with the elevated levels of ELF1 mRNA expression post interferon, suggesting additional mechanisms of post-transcriptional regulation. ELF1 protein levels were increased only in A549 and HeLa cells (Fig 1C, S1C Fig). In contrast, IRF1 protein levels overall showed a more “classical” interferon-dependency: undetectable to low protein levels prior to interferon, and high protein levels post interferon, although this was not observed in PBMC and NHBE (Fig 1A–1C, S1A–S1C Fig). Baseline protein levels in steady-state cells are not unusual for ISG transcription factors. For example, STAT1 and STAT2 proteins are present at baseline and their expression is only slightly enhanced upon interferon-stimulation. What triggers their antiviral activity is activation by phosphorylation [22]. It is possible that steady-state ELF1 also requires activation, and/or that ELF1 functions as part of both the intrinsic (i.e., steady-state) and innate (i.e., induced) immune system.

Finally, we determined the ELF1 mRNA expression response to viral PAMPs. In A549 cells, we found that transfection of the RIG-I ligand poly(I:C) triggered upregulation of ELF1 mRNA expression compared to carrier control (S1D Fig). We found similar results in lung homogenates of mice intranasally challenged with poly(I:C) (S1E Fig). Additionally, polarized human airway epithelial cultures infected with either influenza A virus (IAV) or human parainfluenzavirus 3 (PIV3) showed increased levels of ELF1 mRNA (Fig 1D).

Taken together, we confirmed that ELF1 is an ISG. However, its expression is complex, can be distinct at mRNA and protein levels, and depends on the nature of stimulus, on the timing of treatment, and on the cell type. This was also true for a second transcription factor and well-characterized ISG, IRF1.

### ELF1 exhibits delayed antiviral activity

Our previously conducted screen with > 400 ISGs identified ELF1 as an inhibitor of influenza A virus acting at multi-cycle viral replication [23]. This was unusual, as other reported ISG transcription factors such as IRF1, once expressed, inhibit viruses in the first round of viral replication [3,4]. Thus, we next aimed to validate ELF1’s seemingly delayed antiviral activity.

**Fig 2:**
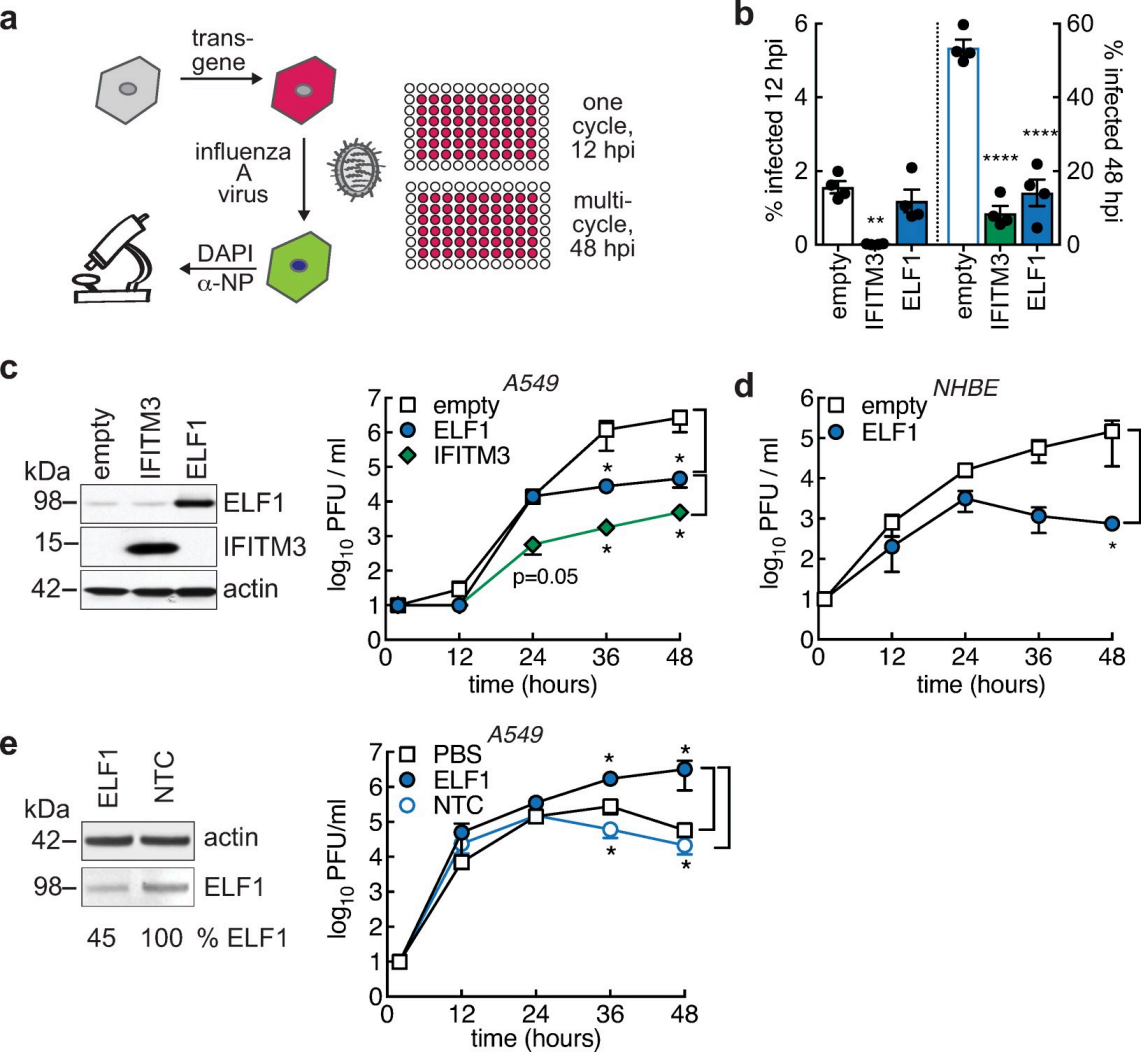
Impact of ELF1 on influenza A virus growth. **a.** A549 were transduced to express transgenes and RFP as control. 48 h post transduction, cells were challenged with a low MOI of influenza A virus, and % of virus–infected (NP–positive) cells determined by high content microscopy after one or multiple replication cycles. **b.** Mean ± SEM of % influenza A/WSN/1933 virus–infected cells by high content microscopy in A549 expressing ELF1, IFITM3 as early (entry) ISG inhibitor control, or empty vector as negative control (n = 4). 12 hpi (one cycle of replication, left y–axis) or 48 hpi (multi–cycle replication, right y–axis). One–way ANOVA and Dunnett’s multiple comparison test versus “empty”. **c.** A549 were transduced to express transgenes ELF1 or IFITM3, or empty vector control. Endogenous and overexpressed ELF1 or IFITM3 protein and actin control post transduction by western blot. Influenza A/WSN/1933 virus growth kinetics on transduced cells (n = 3). Extracellular virus titers by plaque assay on MDCK cells. Individual t–tests between empty and ELF1, or IFITM3 and ELF1, *p<0.1. **d.** Influenza A/WSN/1933 virus growth kinetics on primary normal human bronchial epithelial (NHBE) cells expressing ELF1 or empty vector (n = 3). Extracellular virus titers by plaque assay on MDCK cells. Individual t–tests between empty and ELF1, *p<0.1. **e.** In vivo morpholino oligomers (MO)–mediated ELF1 knockdown in A549. ELF1, ELF1 5’UTR–targeting MO; NTC, 5–base–pair non–targeting mismatch control. Endogenous ELF1 protein and actin control post MO knockdown prior to infection by western blot. % ELF1 protein normalized to actin and mismatch control. Influenza A/WSN/1933 growth kinetics post MO knockdown, n = 3. Mean ± SEM virus titer by plaque assay on MDCK cells. Individual t–tests between PBS control and ELF1 knockdown, or NTC control and ELF1 knockdown, *p<0.1.

First, we used our ISG gain-of-function screening methodology to probe for ELF1 function. We transduced A549 cells to express ELF1 or control genes, challenged the transduced cells with IAV at low MOI for either 12 h (single cycle of IAV replication) or 48 h (multi-cycle IAV replication), then quantified infected cells using immunostaining and microscopy (Fig 2A). High-titer lentiviral stocks ensured even transduction efficiencies, analyzed by scoring RFP reporter-positive cells (S2A Fig). Empty vector served as control for transduction, and IFITM3 was used as an early-acting positive control that blocks IAV fusion during entry. As expected, we found that IFITM3 significantly inhibited infection by influenza A/WSN/1933 (H1N1) virus during single-cycle replication at 12 hpi, and continued to inhibit during multi-cycle replication at 48 hpi (Fig 2B). In contrast, ELF1 exclusively inhibited multi-cycle replication (Fig 2B), recapitulating our previous results [2]. A supplementary high-MOI infection experiment confirmed ELF1’s lack of inhibition during single-cycle replication (S2B Fig). In addition, detailed IAV life cycle studies revealed that ELF1’s delayed antiviral action was not due to inhibition of individual IAV life cycle steps, such as entry, genome replication, egress or infectivity (S3 Fig). Notably, all these assays were performed under single-cycle conditions or while monitoring life cycle steps in isolation. It is possible that ELF1 inhibits IAV at not a single, but at multiple levels during its replication cycle. Alternatively, ELF1 may require post-translational activation in response to a signal elicited by the first cycle of viral replication, thus exclusively affecting subsequent cycles.

In order to determine exactly when ELF1-mediated virus inhibition begins, we assessed low MOI multi-cycle growth kinetics in 12-hour increments on both A549 and primary normal human epithelial cells (NHBE). Viral titers from cells expressing exogenous ELF1 were significantly reduced for both A549 or primary NHBE cells by at least 100-fold compared to empty vector control, starting at 36 or 48 hpi, respectively (Fig 2C and 2D). These results reflect the timing of viral inhibition previously observed by microscopy (Fig 2B). In A549 cells, expression of early inhibitor IFITM3 decreased viral titers earlier, starting at 24 hpi, although the 100-fold difference was not statistically significant until later, at 36 hpi (Fig 2C).

To complement our data obtained by exogenous expression of ELF1, and to probe for ELF1’s contribution to the antiviral response, we used a knockdown approach using in vivo morpholino oligomers (MO). Treatment with MOs designed to bind the 5’UTR of ELF1 mRNA resulted in a 55% reduction of ELF1 protein in A549 cells (Fig 2E left). We measured IAV growth in ELF1-targeting-MO-treated cells compared to both non-targeting-MO-control (NTC)-treated and PBS-treated cells and found increased IAV titers over time (Fig 2E right). This increase was first observed at 36 hpi and was most pronounced at 48 hpi, at which point viral titers were elevated up to 100-fold, consistent with the data obtained by exogenous ELF1 expression (Fig 2C). To verify the specificity of MOs, we knocked down endogenous ELF1 in A549 with the ELF1-MO, then aimed to rescue ELF1’s antiviral function by providing ELF1 wild type as a transgene (S2C Fig). As the ELF1-MO targets the 5’-UTR, it represses the translation of endogenous, but not exogenous ELF1. We visualized IAV infection at 48 hpi by microscopy. As expected, knocking down endogenous ELF1 boosted the number of IAV-infected cells; empty vector control was able to rescue this phenotype (S2D Fig, black and white bars). In contrast, expression of ELF1 provided a functional rescue in ELF1-MO-treated cells (S2D Fig, grey bars). These results validated the specificity of our MO-mediated knockdown.

Together, our data demonstrate that ELF1 is a critical component of the antiviral response *in vitro*, and that its action is most pronounced at multi-cycle virus replication.

### Elf1 is a critical component of the antiviral response to influenza A virus in vivo

**Fig 3:**
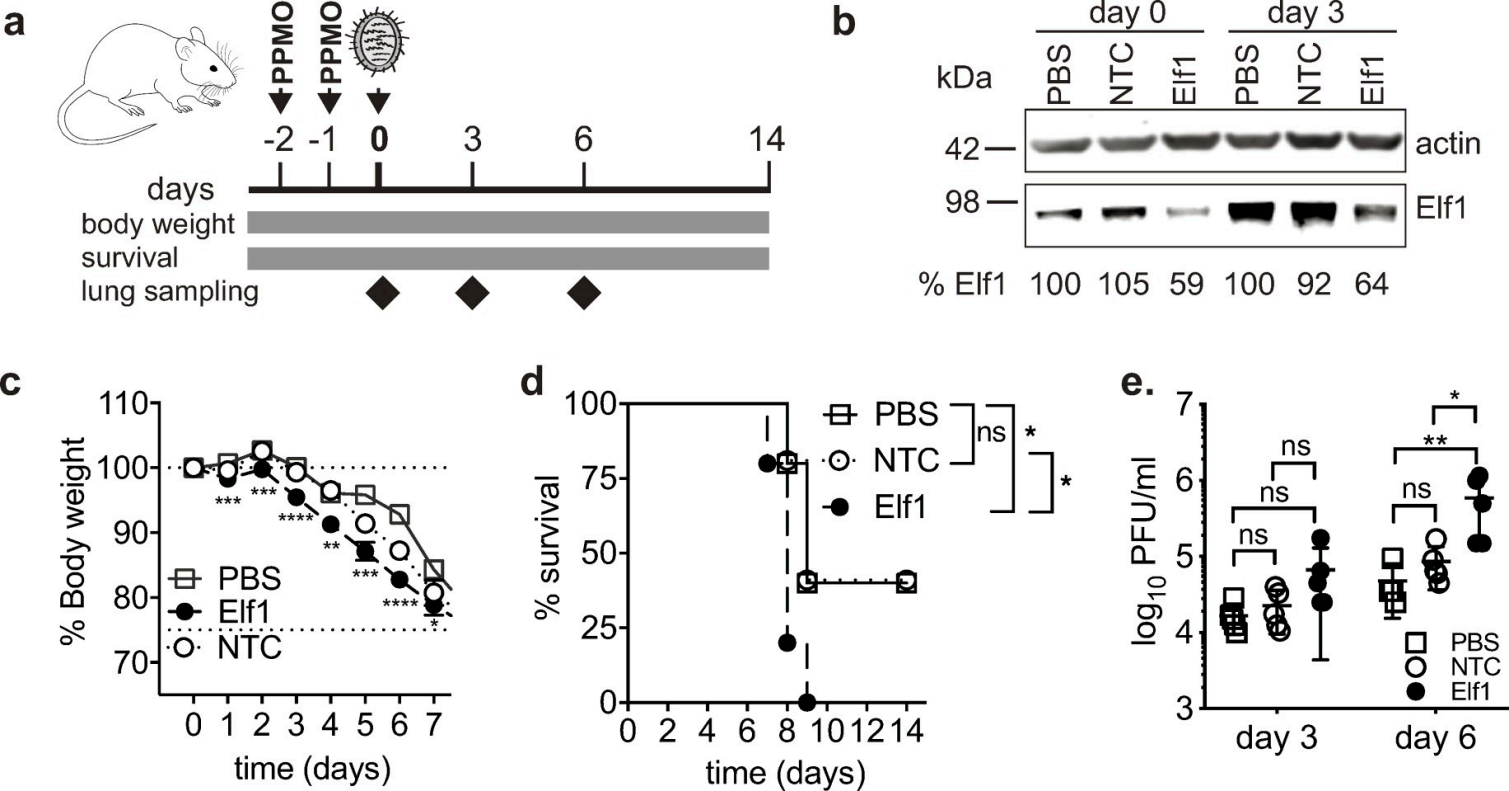
In vivo relevance of Elf1 during influenza A virus challenge. **a.** Schematic of PPMO–mediated in vivo knockdown. PPMOs targeting Elf1, a control mismatch PPMO (NTC), or PBS, were administered to BALB/c mice intranasally at two and one days prior to infection. Mice were then challenged with 40 PFU of influenza A/PR8/1934 virus, and monitored for body weight and survival. Lungs were collected at days 0, 3 and 6 post infection for western blot analyses (**b**) and virus titer determination (**e**). **c.** Mean % body weight ± SEM from PPMO–treated or control mice, n = 15 mice per group. Unpaired two–tailed t–test comparing Elf1 to PBS, *p = 0.1, ** p<0.01,***p<0.001, **** p<0.0001. d. % survival (>25% body weight loss) of PPMO–treated or control mice, n = 5 mice per group. Log–rank Mantel–Cox test, *p<0.1. **e.** Virus titers in mouse lung homogenates were measured by plaque assay on MDCK cells, from n = 5 mice per group. Mann–Whitney test, *p<0.1, **p = 0.01.

Next, we sought to establish Elf1’s relevance *in vivo*, using the mouse model for IAV infection. Previously generated and characterized Elf1^-/-^ mice are viable, but no longer available [17,24]. Therefore, we induced a local knockdown by administering peptide-conjugated phosphorodiamidate morpholino oligomers (PPMO) intranasally, as described previously [25]. PPMOs targeting the 5’UTR of Elf1 and either a non-targeting PPMO mismatch control or PBS, both negative controls, were administered twice prior to IAV challenge (Fig 3A). The Elf1-targeting PPMO yielded approximately 40% *in vivo* knockdown, as determined by quantitative western blot of mouse lung homogenates (Fig 3B). PPMO-treated or control mice were infected intranasally with 40 PFU of influenza A/PR8/1934 (H1N1) virus. Animals with reduced Elf1 lost significantly more body weight and exhibited significantly increased mortality; 100% of Elf1-knockdown animals succumbed to infection, as compared to 50% in either control group. Finally, Elf1-knockdown animals had significantly increased virus titers in the lung (Fig 3C–3E).

This data strongly suggests that Elf1 plays a pivotal role in the antiviral response against IAV *in vivo*.

### ELF1’s antiviral activity relies on intact transcription factor domains

**Fig 4:**
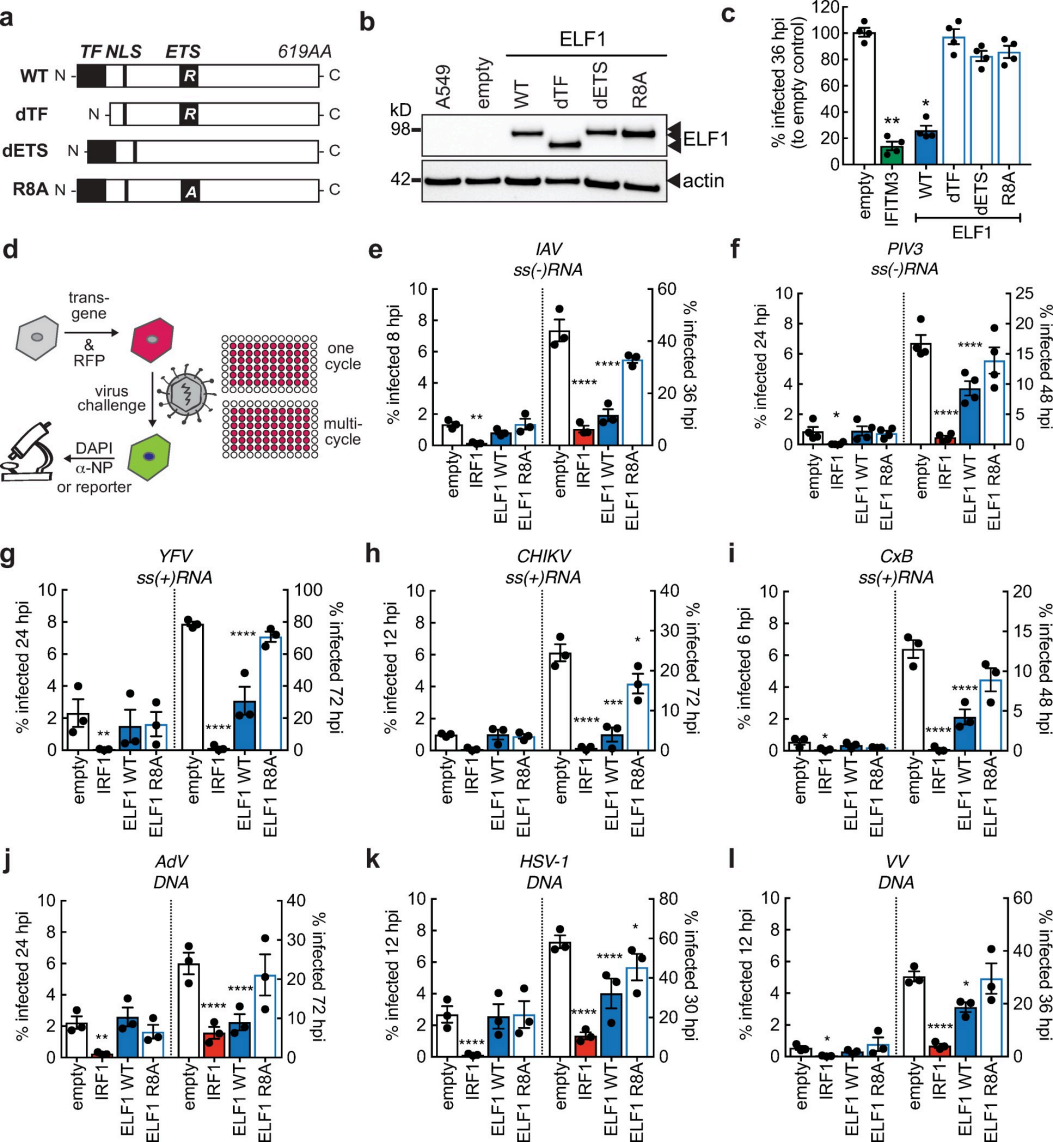
ELF1 impact on multi–cycle replication of diverse RNA and DNA viruses. **a.** ELF1 protein domains and mutational strategy. TF, transcription factor domain; NLS, nuclear localization signal; ETS, E26 transformation–specific domain; R, conserved arginine within DNA binding domain; A, alanine substitution of conserved arginine within DNA binding domain. **b.** Protein expression of mutant ELF1 by western blot. **c.** Mean ± SEM of % influenza A/WSN/1933 virus–infected (NP–positive) cells by high content microscopy in A549 expressing ELF1 wild type, ELF1 mutants, IFITM3 or empty vector (n = 3). One–way ANOVA and Dunn’s multiple comparison test versus empty vector. *p<0.1, **p<0.01. **d.** A549 were transduced to express empty vector as negative control, ISG and transcription factor IRF1 as positive control, ELF1 wild type, or ELF1 R8A, a DNA binding domain mutant. 48 h post transduction, cells were challenged with a low MOI of the indicated viruses and % of infected cells determined by high content microscopy. Mean ± SEM of % virus–infected cells (n = 3) at one replication cycle (left y–axes) or multi–cycle viral replication (right y–axes): **e.** influenza A/WSN/1933 (H1N1), % NP–positive cells, **f.** human parainfluenzavirus 3–EGFP, **g.** yellow fever virus–Venus, **h.** chikungunya–virus–ZsGreen, **i.** coxsackievirus–EGFP, **j.** adenovirus–EGFP, **k.** herpes simplex virus 1–EGFP, or **l.** vaccinia virus–EGFP. YFV (**g**) and CHIKV (**h**) were assayed in the presence of 0.4 μM Ruxolitinib to suppress JAK–STAT signaling and enable virus spread. One–way ANOVA and Dunn’s multiple comparison test versus empty vector of the respective time point, *p<0.1, **p<0.01, ***p<0.001, ****p<0.0001.

ELF1 is a member of the ETS transcription factor family, and has not previously been characterized as an antiviral protein. To test whether ELF1’s antiviral action is mediated through its activity as a transcription factor, we generated ELF1 mutants lacking ETS-transcription factor domains (Fig 4A). These domains were all known or predicted by sequence homology with other related ETS transcription factors [18,26,27]. We deleted either the putative transcription factor (TF) domain predicted to recruit RNA polymerase, or the ETS domain containing the DNA binding domain [27]. Furthermore, within the ETS domain, we substituted an alanine for an arginine (R8) that is conserved and critical for DNA binding in all ETS-transcription factors [26](Fig 4A). At similar transduction efficiencies (S4A Fig), all mutant proteins were present at similar levels as WT ELF1 (Fig 4B). However, mutants were not able to inhibit IAV (Fig 4C), supporting the hypothesis that ELF1 inhibits IAV through its transcription factor activity. From here on, we used the minimal ELF1 DNA binding domain mutant R8A as a negative control in our study.

### ELF1 inhibits multi-cycle replication of diverse RNA and DNA viruses

Thus far, we used IAV as a model virus for characterizing ELF1’s role in the antiviral response. Next, we tested the breadth of ELF1’s antiviral activity against other viruses. We purposefully chose a set of RNA and DNA, enveloped and non-enveloped viruses that represents a wide range of viral replication strategies, regarding entry and genome delivery, transcription initiation, genome replication, protein processing, egress, counteraction of cellular immune responses, and more. We hypothesized that the antiviral action brought on by ELF1 might affect these viruses differently, which could shed light on ELF1’s mechanism of inhibition. We determined the effect of ELF1 on single- and multi-cycle replication of eight viruses (Fig 4D-l and S5 Fig): influenza A/WSN/1933 (H1N1) virus (IAV), human parainfluenzavirus 3 (HPIV3), yellow fever virus (YFV), chikungunya virus (CHIKV) (all three enveloped +RNA viruses), coxsackie B virus (CxB, a non-enveloped +RNA virus), herpes simplex virus 1 (HSV-1), vaccinia virus (VV) (both enveloped DNA viruses), and adenovirus 5 (AdV, a non-enveloped DNA virus). Empty vector-transduced cells served as control for the effects of transduction, and ELF1 R8A-expressing cells as a control for ELF1 protein devoid of its transcription factor activity (Fig 4C). The ISG and transcription factor IRF1 served as positive control, as it is known to inhibit all of these viruses in single-cycle assays [3,4]. A549 were transduced with high-titer lentiviral stocks to generate similar levels of transduction between samples (S4B–S4I Fig, S5 Fig), and consequent expression of ELF1 or controls did not cause measurable cytotoxicity (S6 Fig). The differences in replication strategies among these viruses are reflected by different replication rates, which we considered when designing our single- and multi-cycle assays (Fig 4E-l, time points indicated at bottom of x-axes). We found that both IRF1 and ELF1 significantly inhibited all viruses in the panel (Fig 4E-l and S5 Fig), indicating that the breadth of ELF1-mediated virus inhibition is similar to that mediated by IRF1 and the immediate interferon response. However, and as seen in previous experiments, one notable difference between IRF1 and ELF1 was in timing, as ELF1 inhibited all viruses exclusively at multi-cycle replication (Fig 4E-l and S5 Fig). Interestingly, this multi-cycle antiviral action was apparent irrespective of the virus life cycle length (ranging from 6 to 24 h for a single cycle). Therefore, it is possible that inherent differences in cells being challenged for the first time versus being challenged repeatedly contribute to ELF1’s delayed antiviral activity. Of note, YFV and CHIKV are both sensitive to endogenous interferon in A549 cells and were thus assayed in the presence of a JAK1/2 inhibitor Ruxolitinib to suppress JAK-STAT signaling and allow for viral replication (Fig 4G and 4H, S5C and S5D Fig and S7 Fig). ELF1 inhibited both YFV and CHIKV in the presence of Ruxolitinib.

Taken together, these results show that ELF1 does not affect the tested viruses differently, as it has, in fact, broad antiviral activity. Further, our data demonstrate that ELF1, when expressed exogenously, does not require JAK-STAT-signaling to inhibit viruses.

### ELF1’s antiviral mechanism does not act through induction of canonical interferon signaling

**Fig 5:**
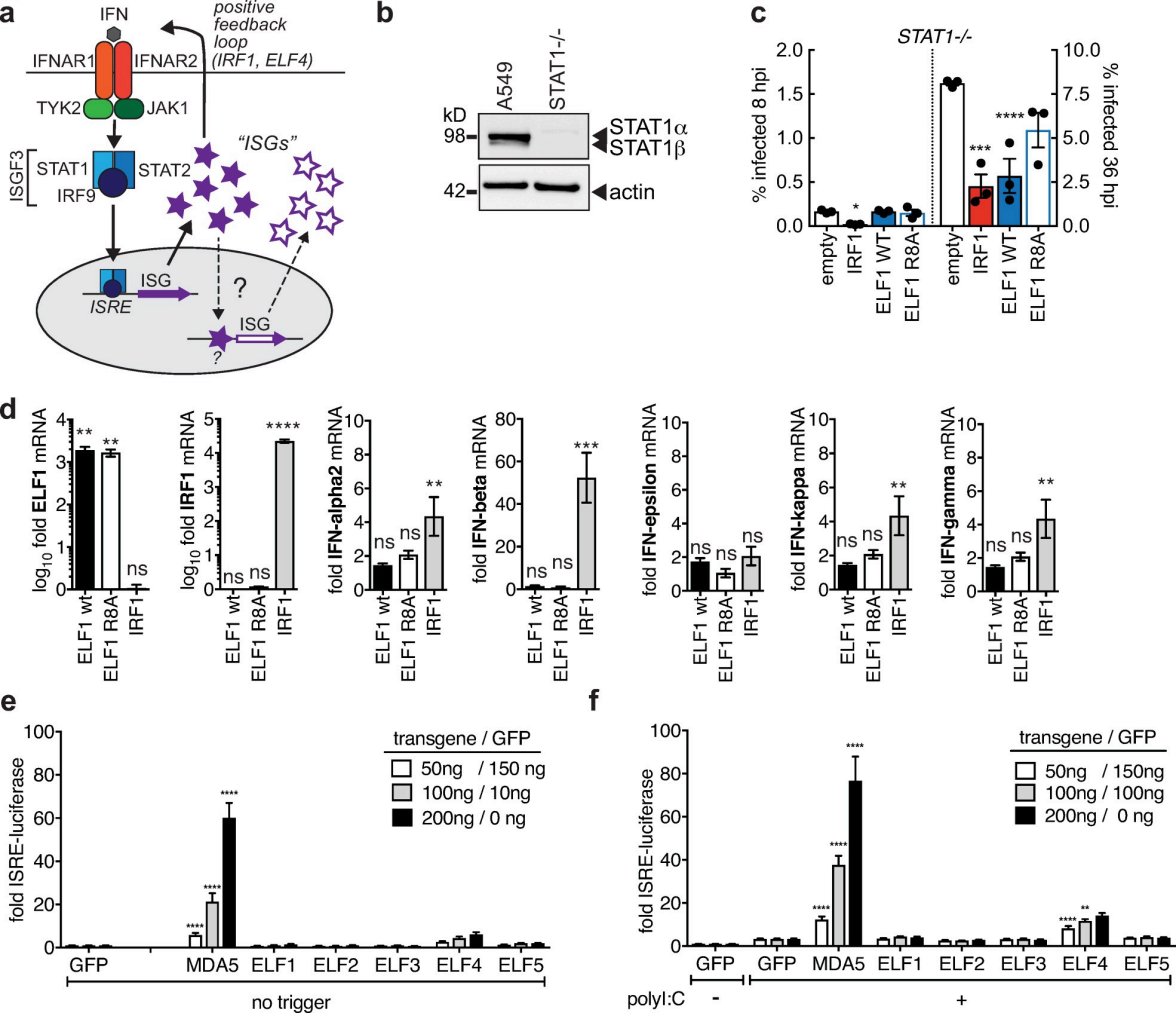
ELF1 downstream impact on interferon production and signaling. **a.** Canonical interferon (IFN) signaling (solid arrows) and proposed second wave of ISG expression (dashed arrows). **b.** STAT1 protein expression in A549 STAT1^–/–^or control cells by Western blot. **c.** Mean ± SEM of % influenza A/WSN/1933 virus–infected (NP–positive) cells by high content microscopy in STAT1^–/–^A549 expressing ELF1, ISG and transcription factor IRF1 as positive control, or empty vector (n = 3). ANOVA with Dunnett's multiple comparison to empty. ****p<0.0001, ***p<0.001 *p<0.1. **d.** A549 transduced to express ELF1 wild type (wt), ELF1 loss–of function mutant R8A, IRF1 as positive control, or empty vector control. mRNA of type I and II interferons by RT–qPCR. Data is shown normalized to empty control, as mean ± SEM. ****p<0.0001, **p<0.01. **e.** Reporter assay for ISRE–driven transcription. 293T encoding firefly luciferase under the control of a promoter carrying the ISRE motif and stably expressing renilla luciferase as control was transfected to express GFP as negative control, MDA5 as positive control, or ELF1, ELF2, ELF3, ELF4 and ELF5, respectively. Data as mean ± SEM from n = 3 independent experiments. **f.** 293T luciferase reporter cells were treated as in (e), and subsequently stimulated by transfection of polyI:C. Data as mean ± SEM from n = 3 independent experiments.

Thus far, we have shown that ELF1 is an ETS transcription factor with broad antiviral activity, which seems to act exclusively on multi-cycle viral replication. Such temporal divergence could theoretically occur through a second round of canonical interferon signaling in the form of positive feedback mechanisms (Fig 5A, solid arrows). An example of this mechanism is demonstrated by ELF4, which exerts its antiviral function through feeding-forward to produce more interferon [18]. Another example of such a positive feedback loop is seen in the ISG IRF1, which takes a two-pronged approach to viral inhibition by triggering the production of interferon, as well as regulating its own set of antiviral ISGs [28]. Thus, we postulated that ELF1 acts through the induction of canonical interferon signaling, similar to IRF1 and ELF4.

To test this, we first performed IAV single- and multi-cycle replication assays expressing ELF1 or positive control IRF1 on A549 lacking STAT1 (Fig 5B, S8 Fig), a critical component of ISGF3 [8](Fig 5A). This experiment tested for STAT1-dependency of ELF1 downstream function, as the ELF1 protein was expressed exogenously. In line with our results using Ruxolitinib to block the action of JAKs (Fig 4G and 4H), we found that exogenously expressed ELF1 retained its antiviral activity in the absence of STAT1, as did IRF1 (Fig 5C). However, there was a striking difference in timing between the effects of IRF1 and ELF1: IRF1 inhibited IAV after one cycle of viral replication (as previously reported [3]), while ELF1, again, inhibited IAV exclusively in multi-cycle replication. This suggests that ELF1 downstream antiviral function is not through the induction of canonical interferon signaling.

To corroborate this finding, we directly tested whether ELF1 induces expression of type I or II interferons by RT-qPCR. In contrast to IRF1 control, which induced expression of interferon alpha, beta, kappa and gamma, ELF1 did not induce expression of any tested type I or II interferons (Fig 5D). We thus determined that ELF1 might not exert its downstream antiviral function through a positive interferon feedback loop.

In a parallel approach, we performed ISRE reporter assays to test whether ELF1 induces gene expression from the ISRE element, the regulatory element recognized by ISGF3 [29]. Other human ELF family members (2, 3, 4 and 5) were also tested; MDA5 served as positive, and GFP as negative control (Fig 5E and 5F). We found that, in contrast to MDA5 and ELF4 [18], ELF1 indeed does not induce transcription from the ISRE reporter.

Taken together, we conclude that ELF1 does not inhibit viruses through initiation of STAT1-dependent interferon signaling, nor through production of type I interferons. Consequently, induction of another round of interferon signaling is not the mechanistic basis for ELF1’s delayed mode of antiviral action.

### ELF1 results in expression of a vast transcriptional program that is distinct from both the early and the late interferon response

**Fig 6:**
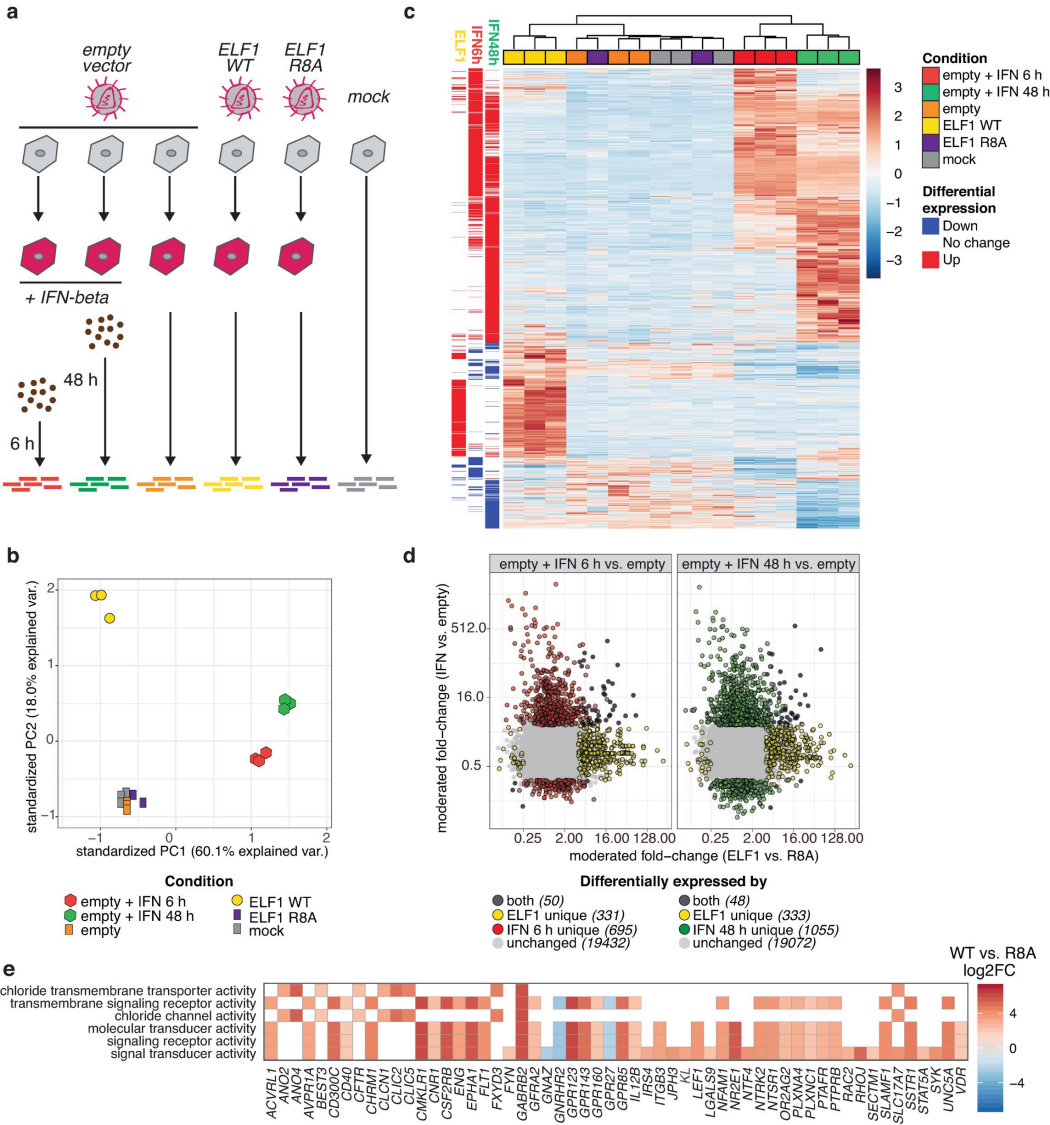
ELF1 transcriptional program. **a.** Schematic of RNA–Seq approach. A549 cells were mock–transduced or transduced to express empty vector, ELF1 wild type (WT), or ELF1 loss–of–function mutant R8A, respectively. At 48 h post transduction, two empty–transduced samples were treated with 500 U/ml of IFN–beta for 6 or 48 h, respectively. RNA was harvested and analyzed by RNA–Seq. All samples were generated from n = 3 independent biological experiments (one ELF1–R8A replicate was excluded from analysis due to technical problems). **b.** Principal component analysis of 1000 most variable genes across all samples (regularized log transformed counts, after correction for replicate batch effects). **c.** Heatmap plotting z–scaled expression values for genes differentially expressed (adjusted p–value < 0.05, log_2_ fold–change >2 or <–2) in either of the interferon–beta contrasts (compared to untreated, empty–vector transduced control) or both of the ELF1 contrasts (ELF1–WT vs ELF1–R8A, and ELF1–WT vs empty vector). Hierarchical clustering (complete method) was performed on Euclidean distance values. Sidebar annotation labels indicate differential expression (up regulation, red /down regulation, blue) for listed contrasts. **d.** Scatter plots of moderated log_2_ fold–change values for all expressed genes evaluated in differential expression analyses. Points (genes) are colored according to differential expression (adjusted p–value < 0.05, log_2_ fold–change >2 or <–2) in the indicated pairwise contrasts. **e.** Enriched GO terms (Molecular function ontology, adjusted p value < 1.0 x 10^−6^) and constituent genes in ELF1–WT differentially expressed genes.

To determine the transcriptional program regulated by ELF1, we performed differential gene expression analysis by RNA-seq. We aimed to use A549 cells lacking ELF1 as a comparative control; however, generating a viable clonal A549 ELF1^-/-^ knockout line by CRISPR/Cas9 genome editing proved challenging. Thus, we instead chose a tightly controlled overexpression strategy to pinpoint the mechanism of the ELF1 antiviral program. First, we established negative controls: empty vector-transduced and mock-transduced A549 were used to control for effects of transduction (Fig 6A), and ELF1 R8A to control for the effect of ELF1 protein devoid of transcription factor activity (Fig 6A). Principal component analysis revealed that mock-transduced, empty vector-transduced, and ELF1 R8A-transduced A549 were transcriptionally more similar to each other than to ELF-transduced or interferon-stimulated samples, thereby validating our controls (Fig 6B).

Next, we aimed to identify genes whose expression changes upon ELF1 wildtype (WT) expression. We performed differential gene expression analyses of mock-, empty vector-, ELF1 WT-, or ELF1 R8A-transduced controls. Ectopic expression of ELF1 WT significantly altered the expression of 381 genes, most of which (353) were upregulated relative to control conditions (Fig 6C, S1 Table). The size of the differentially expressed gene list and the previously observed potency and broadness of ELF1’s antiviral protection are reminiscent of the interferon response.

Hence, we next aimed to examine how different the ELF1 differentially expressed program was from the interferon response. Historically, the term “interferon-stimulated gene” has been defined loosely as a gene whose mRNA expression changes post-interferon stimulation. This classification has been used irrespective of the cell type, fold upregulation, and time post-interferon-stimulation [30]. Given ELF1’s delayed mode of antiviral action, we focused our analysis on the temporal aspect of the interferon response by comparing gene expression in A549 transduced with ELF1 WT to gene expression in A549 (transduced with empty vector) treated with interferon-beta at early (6 h) and late (48 h) time points (Fig 6A). We first identified early (S2 Table) or late (S3 Table) ISGs by differential expression testing (Fig 6C). Consistent with previous studies [30,31], we found some overlap between early and late ISGs, but a large number of ISGs were unique to the early or to the late response (Fig 6C, S2 Table, S3 Table). Thus, it is clear that specific ISGs are upregulated at different time points post-interferon exposure. Most ISGs (early or late) were largely distinct from those genes identified as differentially expressed by ELF1 WT expression (Fig 6B and 6D). Indeed, further differential expression analysis of the interferon response (interferon 6 h or 48 h *vs*. mock treated) compared to the ELF1 WT program (ELF1 WT-transduced *vs*. ELF1 R8A-transdued) identified more than a thousand genes with significantly distinct expression patterns (S4 Table, S5 Table). We concluded that, under the experimental parameters examined, the transcriptional program elicited by the ETS transcription factor ELF1 is distinct from the interferon transcriptional response. Furthermore, exogeneous ELF1 expression did not trigger significant induction of interferon type I, II or III genes, or other inflammatory cytokines such as TNF or IL-6 (S9 Fig, S1 Table). These findings corroborated our previous results from RT-qPCR and ISRE reporter assays (Fig 5D–5F), but were contrary to a previous study that found ELF1 to enhance the transcriptional response to interferon-beta [32]. The differences might indicate cell-type specific differences between HeLa cells [32] and A549 cells in the present study.

To begin exploring possible mechanisms for ELF1-mediated antiviral effects, we performed gene ontology (GO) enrichment analysis on the ELF1 differentially expressed genes identified by RNA-Seq. Many of the most significantly enriched GO terms in the Cellular Component and Molecular Function categories relate to the cell membrane and/or receptors (Fig 6E, S9 Fig, S6 Table). This list of genes includes G-protein-coupled-receptors (e.g. *GPR123*), cytokines (e.g. *IL12B*), and genes involved in chloride transport (e.g. *GABRB2*). Of note, genes differentially expressed upon ELF1 WT expression were not enriched for GO terms implicated in IAV egress or infectivity, such as cargo receptor activity, membrane-to-membrane docking, or proteolysis (S6 Table), which is in line with our results indicating that the ELF1-mediated antiviral program likely does not target individual steps of the IAV life cycle.

Taken together, we find that ELF1 expression elicits a vast transcriptional program that is distinct from both the early and the late interferon response. Although GO analyses should be interpreted with some caution, our findings open up the possibility that ELF1 acts through an additional round of antiviral signaling, which might explain ELF1’s delayed antiviral phenotype.

### ELF1 upregulates bona fide target genes, the majority of which are involved in cell signaling

**Fig 7:**
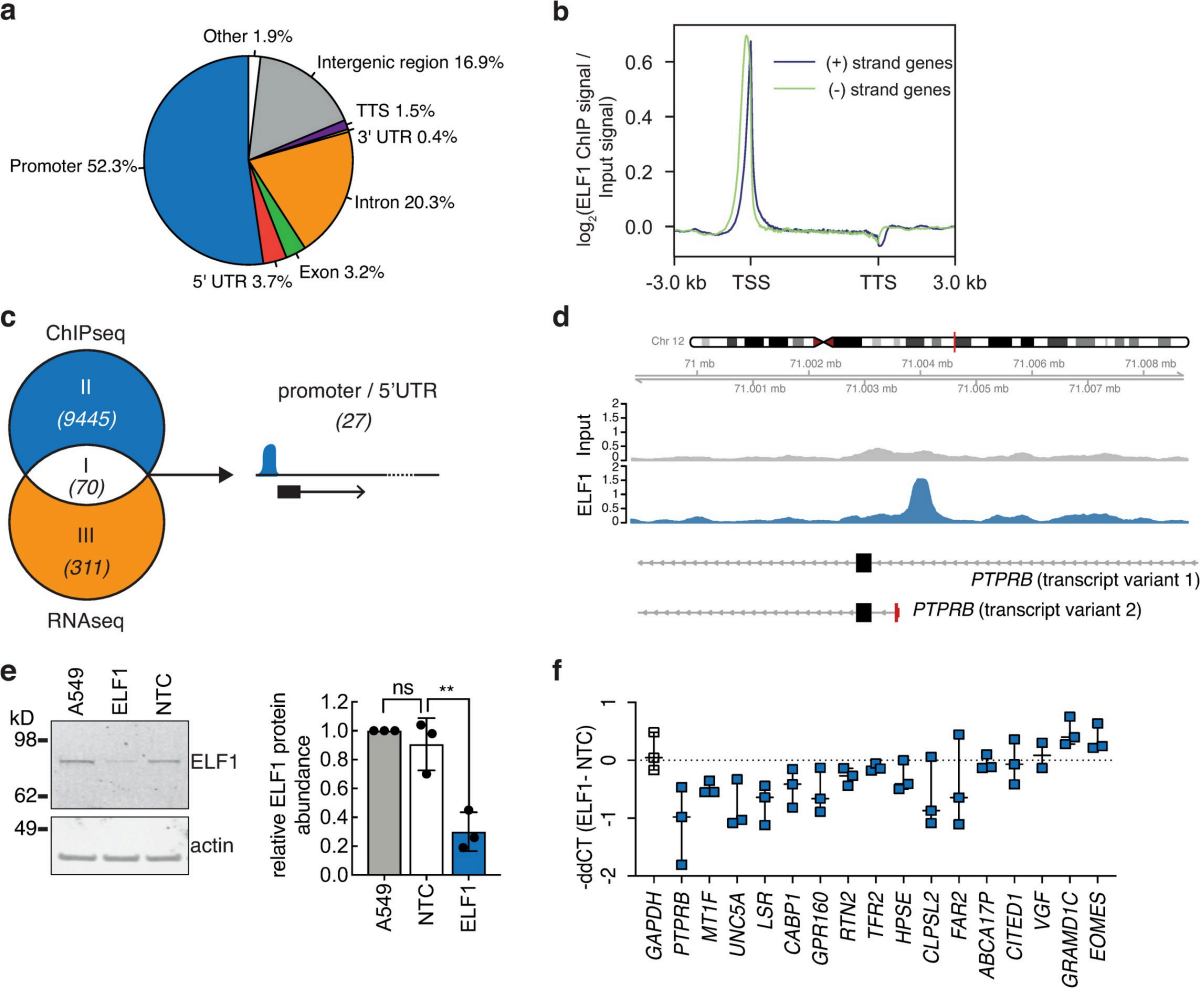
ELF1 genomic occupancy and validation of ELF1 target gene expression. **a.** A549 cells were subjected to ChIP–seq using anti–ELF1 antibody. Pie chart visualizes the distribution of ELF1 peaks over promoters, untranslated regions (UTR), introns, exons, transcription termination sites (TTS), and intergenic regions. **b.** Representative Meta–gene plot of average log2 enrichment of ELF1 ChIP signal (reads per million mapped reads) over input control signal (reads per million mapped reads). Genes were normalized to a length of 5 kb, then the average log2 ChIP signal over input signal was plotted over the meta gene including 3 kb upstream and downstream of the transcription start site (TSS) and transcription termination site (TTS). Blue line shows average log2 ChIP enrichments over positive–strand genes and green line shows enrichment over negative–strand genes. **c.** Venn diagram depicting the overlap of ELF1 differentially expressed genes (ELF1 WT vs. (ELF1 R8A + empty + mock–transduced), Fig 6, RNA–seq) and genes with at least one ELF1 binding site in regions depicted in panel (a). Number of genes shown in brackets. **d.** Representative ChIP–seq track of ELF1–enrichment in the promoter region of one of the 27 genes noted in (c).Y–axis represents reads per million mapped reads spanning a genomic position. Transcription start site (TSS) for PTPRB (transcript variant 2) is highlighted in red and genomic coordinates ± 5 kb around TSS are noted above. **e.** A549 cells were treated with 50 μM of MOs for 24 h and knockdown efficiency determined by Western blot analysis. Protein levels quantified by densitometry, normalized to actin control and A549 untreated control, shown as mean ± SEM from n = 3 independent experiments. One–way ANOVA and Dunnett’s multiple comparison test versus NTC, **p = 0.023. ELF1, cells treated with ELF1–targeting MO, NTC, cells treated with non–targeting MO control, A549, mock–treated cells. **f.** A549 cells were treated with 50 μM of MOs for 24 h, and mRNA expression of candidate ELF1 target genes or GAPDH as negative control determined by RT–qPCR normalized to housekeeping gene RPS11.–ddCT (normalized CT value of ELF–targeting MO treated cells–normalized CT value of non–targeting MO treated cells). Data shown as n = 3 independent biological replicates; box–and–whiskers bars show range and mean.

The transcriptional changes brought upon by ELF1 WT expression, abolished upon disturbance of its DNA binding domain in the R8A mutant, are consistent with ELF1’s role as a transcription factor. To modulate gene expression, transcription factors may act at target promoters or at enhancer regions, or both. To investigate how ELF1 may be regulating target genes, we performed ChIP-seq analysis in A549 cells, measuring endogenous ELF1 DNA binding at steady-state on a genomic scale. We found that while ELF1 did bind distal intergenic sites, the majority of ELF1 peaks were present in promoter regions, close to transcription start sites (Fig 7A and 7B), suggesting that ELF1 may regulate genes primarily through promoter proximal effects and rather than through distal effects. Hence, we concluded that our ChIP-seq list of ELF1-promoter-bound genes likely contains genes whose expression is directly regulated by ELF1.

Promoter binding does not always correlate with a change in gene expression. To link expression changes of individual genes and ELF1 promoter binding, we mined our ChIP-seq and RNA-seq data sets for genes that have both a proximal ELF1 binding peak and are differentially regulated upon ELF1 WT expression (Fig 7C). ELF1 bound at promoters, intragenic, and/or intergenic regions in 70 of the 381 differentially expressed genes (Fig 7C, Venn diagram region I; representative ChIP-seq track shown in Fig 7D; S7 Table, S8 Table). Of the 70 genes, 27 genes showed ELF1 binding within 1 kb of their transcription start site. The relatively low number of RNA-seq/ChIP-seq overlapping genes could reflect the different sensitivities of the two methods, the fact that ChIP-seq captures protein-coding and non-coding genes (whereas our RNA-seq strategy only captures protein-coding genes), and/or of different cutoffs used during the analysis. Biologically, it could mean that some genes are bound by ELF1 but do not exhibit a significant expression change (Fig 7C, Venn diagram region II), and that other, differentially expressed genes are regulated by ELF1 indirectly (Fig 7C, Venn diagram region III).

To individually validate direct ELF1 target genes, i.e. genes that are regulated by ELF1 through binding at their promoters, we knocked down ELF1 with MOs (Fig 7E) and analyzed gene expression changes of candidate genes by RT-qPCR (Fig 7F). From 27 candidate genes, we were able to design specific primers for 21, and 16 genes were expressed to detectable levels in steady-state A549. The majority of these genes showed reduced mRNA expression upon ELF1 knockdown (Fig 7F), suggesting a direct functional role of ELF1 in the transcription of these genes. This functional validation, together with results from ChIP-seq demonstrating ELF1 promoter binding, led us to conclude that these genes are bona fide direct ELF1 target genes. The target genes encode enzymes (Fatty Cycl-CoA Reductase 2, *FAR2*; Heparanase, *HPSE*), a protein involved in endoplasmic reticulum vesicle transport (Reticulon 2, *RTN2*), a protein associated with metal ion transport (Metallothionein 1F, *MT1F*), a protein with unknown function (Colipase-like protein 2, *CLPSL2*), multiple receptors (Protein Tyrosine Phosphatase Receptor Type B, *PTPRB*; Unc-5 Netrin Receptor A, *UNC5A*; Lipolysis Stimulated Lipoprotein Receptor, *LSR*; G Protein-Coupled Receptor 160, *GPR160*; Transferrin Receptor 2, *TFR2*), and a protein involved in calcium-ion-mediated cellular signal transduction (Calcium-binding protein 1, *CABP1*; Fig 7F).

In summary, we show that the ETS transcription factor ELF1 regulates a complex transcriptional program that is distinct from both the early and the late interferon response. ELF1 exhibits delayed antiviral activity against diverse RNA and DNA viruses. ELF1 differentially expressed genes relate to the cell membrane and/or receptors. Finally, ELF1 directly upregulates bona fide target genes, the majority of which are involved in cell signaling. Based on these observations, we hypothesize that ELF1 achieves antiviral function through direct regulation of genes that provide an additional round of immune signaling.

## Discussion

In this study, we establish the ETS transcription factor ELF1 as a previously unrecognized, yet critical antiviral regulator *in vitro* and *in vivo*.

Previous reports exclusively characterized functions of murine Elf1 in lymphocytes. Elf1 is a mediator of T-cell antigen receptor expression and regulator of natural killer cell maturation [27,33,34]. It induces IL-2 expression and feeds into the MAPK pathway in various lymphocytes [27,33]. It is possible that the *in vivo* antiviral activity of Elf1 that we report is mediated by these mechanisms, and thus by cellular immunity. However, our *in vitro* data on lung epithelial cells support a role for ELF1 that is cell-intrinsic and lymphocyte-independent. Such different roles in specific cellular contexts are known for a number of other innate immune transcription factors. For example, the transcription factor IRF1 has been shown to both support the development of CD8^+^T and natural killer cells [35,36], and to raise a cell-intrinsic antiviral program by activating interferon- and ISG-expression in fibroblasts, epithelial cells and skeletal muscle cells [12,20,37,38]. It is possible that ELF1 similarly displays multiple functions depending on the type and transcriptional state of cells.

We demonstrate that ELF1 is present in many cell types *in vitro* at steady-state. In some cell types, but not all, its expression is further upregulated upon interferon stimulation. Whether upregulation is a prerequisite for ELF1’s antiviral function, i.e. whether ELF1 is part of the intrinsic (steady-state) or the innate (interferon-regulated) antiviral response, is unclear; it may be both. One mechanism of interferon-dependent function could be through posttranslational modification, which would repurpose the modified ELF1 for a new, antiviral role. However, we have not yet found any evidence for biochemical differences between basal ELF1 and interferon-regulated ELF1. Another mechanism could be a model of “kinetic control” of transcription factor function. This term was first coined in association with the transcription factor IRF4: upon reaching a critical threshold of IRF4 expression, low-affinity IRF4 binding sites recruit IRF4, and the subsequent changes in transcription determine a functionally different outcome [39]. It remains to be determined if a similar critical threshold exists for ELF1. Upon interferon-mediated upregulation, ELF1 may saturate new low-affinity binding sites, which may allow for regulation of genes that contribute to ELF1’s antiviral activity.

One challenge of our study was to define specific antiviral mechanisms brought on by ELF1’s diverse transcriptional program, which was reminiscent of the challenges of characterizing the antiviral mechanisms of ISGs [1]. Our data establish that ELF1’s antiviral mechanism is through the regulation of genes. We found that ELF1 may regulate genes both directly (i.e. through binding to their promoters) and indirectly (i.e. through induction of an additional round of antiviral signaling). Evidence for indirect gene regulation by ELF1 are the relatively low overlap between genes that are upregulated upon ELF1 expression and genes that have ELF1 bound at their promoters, the fact that top ELF1 differentially expressed genes fall into signal transduction categories, and our finding that the majority of bona fide direct ELF1 target genes are involved in signaling processes. Interestingly, some ELF1 (direct and putative indirect) targets act in conjunction with ion channel activity. Cell-to-cell communication by stimulation of cell surface receptors which relay the stimulus to induce the flux of ions has been shown to regulate a variety of stress responses [40]. A recent study unraveled a connection between ion channel activity and innate antiviral signaling pathways. Stromal interaction molecule 1 (STIM1), a Ca^2+^ sensor, negatively regulates the type I interferon response by retaining the adaptor molecule stimulator of interferon genes (STING) at the endoplasmic reticulum [41]. Thus, loss-of-function mutants in *STIM1* may induce autoinflammatory phenotypes. Another protein regulated downstream of STIM1 is PTPRB [42], which we identified as an ELF1 target. This may suggest involvement of ELF1 in the STIM1-STING signaling axis.

However, and distinct from its relative ELF4 [18], we confirmed that ELF1 does not act through feeding-forward to produce interferons. In fact, we show that ELF1 adds a layer of antiviral protection that is distinct from both the early and the late type I interferon response. What is the evolutionary rationale for multiple antiviral gene expression programs? One answer could be to provide multiple layers of antiviral mechanisms to cover antiviral strategies for as many diverse viruses as possible [4]. Indeed, functional redundancy is a common theme of the innate immune response [43]. Another answer could be to mitigate adverse effects brought about by prolonging existing ones. The multi-layered antiviral state raised by interferon represents a double-edged sword, as the associated inflammation can have detrimental effects [44]. This is exemplified by genetic defects in key mediators of innate immune signaling that ultimately cause interferon overproduction or failure to return cells to homeostasis post-interferon exposure [44]. Interestingly, multiple independent genome-wide association studies found single nucleotide polymorphisms in the ELF1 open reading frame or in ELF1 target DNA binding sites to be associated with chronic inflammatory disorders such as Crohn’s disease, inflammatory bowel disease, and systemic lupus erythematosus [45–51]. How ELF1 may contribute to excess inflammation in these disorders remains elusive. However, transcriptional dysregulation, e.g. of interferon- or NFκB-mediated programs, has been shown to contribute to disease pathogenesis [52–57]. These findings and ours raise the possibility that ELF1 fulfills a major regulatory function both in inflammation and in antiviral immunity.

While the aforementioned studies have begun to untangle the complexity of innate transcriptional programs, they also add to our growing knowledge of temporal gene expression dynamics in the innate immune response. Using novel transcriptional profiling techniques, several recent studies reveal that genes downstream of interferon signaling can be classified into qualitatively distinct modules with different temporal expression dynamics post-interferon [31,58,59]. How temporal divergence may influence the antiviral potency of the interferon response, and which factors or pathways drive divergent gene expression dynamics, remain unknown. In fact, a puzzling detail about ELF1 remains why it exclusively inhibits multi-cycle viral replication. This phenotype sets ELF1 apart from other known innate transcription factors such as IRF1 or ELF4 [4,18]. It is possible that the part of ELF1-mediated gene expression that is critical for antiviral activity is indeed downstream of an additional signaling cascade, which simply requires more time to raise an antiviral barrier. While our data support the presence of such an additional signaling cascade, we argue that this is unlikely to be the (only) reason for ELF1’s delay, as other transcription factors that induce interferon signaling, and are expressed prior to a virus challenge, inhibit viruses immediately [4,18]. An alternative explanation is that ELF1 has a differential role in naïve versus virus-infected cells. Thus, we propose that a first round of viral infection is required for ELF1 to protect cells from subsequent rounds of infection. A molecular mechanism for such a switch might be through interaction of ELF1 with a protein that is only present (or active) upon viral infection. This putative partner would help ELF1 relocate to regulatory regions of specific antiviral genes. This dynamic model fits our observation that ELF1 inhibits different viruses uniquely at multi-cycle replication irrespective of the virus life cycle length and would provide an attractive new mechanism for temporal divergence of antiviral programs.

Defining alternative antiviral transcription programs such as that raised by ELF1 may pave new avenues in rational drug design. Transcription factors have historically been considered to be undruggable, but this paradigm is slowly shifting [60]. Novel small molecules are being developed that mimic DNA binding properties [61] or disrupt protein-protein interactions critical for transcription factor function [62], and artificial ligands aim to modulate transcription factor activation [63]. There is an unmet need for novel antiviral drug targets, especially to combat emerging viruses, recently exemplified by Zika and Ebola viruses [64,65]. ELF1 inhibits every virus we have tested in this study, including members from diverse (-)RNA, (+)RNA, and DNA virus families. Thus, harnessing the antiviral power ELF1 could be an attractive approach for broadly antiviral therapies.

## Materials and methods

Detailed information on materials, including sources, are listed in S9 Table and S10 Table.

### Contact for reagent and resource sharing

Further information and requests for resources and reagents should be directed to and will be fulfilled by the Lead Contact, Meike Dittmann (Meike.Dittmann@nyumc.org).

### Ethics statement

Buffy-coats obtained from anonymous blood donors were obtained from the New York Blood center. Whole blood was obtained from healthy and de-identified donors that signed an informed consent. All research studies involving the use of animals were reviewed and approved by the Institutional Animal Care and Use Committees of the Icahn School of Medicine at Mount Sinai, and were carried out in strict accordance with the recommendations in the Guide for the Care and Use of Laboratory Animals (IACUC-2017-0330).

### Animals

Five-week-old female BALB/cJ mice were purchased from Jackson Laboratories (stock number 000651). 6-week-old C57/BL6 mice were bred in-house.

### Primary human cells

PBMC were isolated from blood using a Ficoll-Paque PLUS (GE Amersham) gradient [66] and maintained in RPMI (Invitrogen). PBMC were used for functional studies.

Primary human normal airway tracheobronchial epithelial cells from de-identified donors (NHBE) (sex: male and female) were provided by Lonza (Walkersville, MD) and grown in BEM media supplemented with the BEGM bullet kit (Lonza). NHBE were used for functional studies, and for generation of polarized human airway epithelial cultures (HAE). To generate HAE, NHBE from individual donors were expanded on plastic to generate passage 1 cells, which were subsequently plated (5x10^4^ cells/well) on rat-tail collagen type 1-coated permeable transwell membrane supports (6.5mm; Corning Inc). HAE cultures were grown in B-ALI medium supplemented with inducer (Lonza Inc.) at each media change with provision of an air-liquid interface for approximately 6 weeks to form differentiated, polarized cultures that resemble *in vivo* pseudostratified mucociliary epithelium.

### Cell lines

A549 (adenocarcinomic human alveolar basal epithelial cells, human; sex: male; ATCC), A549 CRISPR STAT1^-/-^ (Laboratory of Adolfo Garcia-Sastre), HeLa (cervix epithelial cells; human; sex: female; ATCC), HFF (human foreskin fibroblast cells; human; sex: male; ATCC), 293T (embryonic kidney epithelial cells; human; sex: female; ATCC), 293T LentiX (Clontech Laboratories), 293T ISRE reporter (Laboratory of Adolfo Garcia-Sastre), MDCK (kidney epithelial cells; canine; sex: female; Laboratory of Wendy Barclay), LLC-MK2 (kidney epithelial cells; rhesus macaque; Laboratory of Wendy Barclay), and Vero (kidney epithelial cells; African green monkey; sex: female; ATCC) cells were maintained in DMEM (Invitrogen) supplemented with 10% fetal bovine serum (FBS), 1% NEAA, 1% P/S. A549 and HFF were used for virus infection and functional studies. MDCK, HeLa, LLC-MK2 and LentiX cells were used for virus production and virus titration. All cell lines were obtained directly from the ATCC (with exceptions of Lenti-X 293T cells, which were obtained from Clontech Laboratories, and MDCK and LLC-MK2 cells, which were obtained from the laboratory of Wendy Barclay). All cell lines were grown at 37°C, individually expanded, and all seed and working stocks tested negative for contamination with mycoplasma. Cells were used in experiments below passage 15 from thaw, or when population doubling times slowed beyond 25% of seed stock doubling times.

### Viruses

Influenza A/WSN/1933 (H1N1) virus stock was grown in MDCK cells. Influenza A/California/04/2009 (H1N1) virus stock, grown in embryonated chicken eggs, was obtained by BEI resources. The following virus stocks were grown as previously described: HPIV3-GFP (based on strain JS) on LLC-MK2 cells [67], CxB-GFP (based on pMKS1-GFP) on HeLa cells [68], YFV-Venus (YF17D-5′C25Venus2AUbi) on Vero cells [69], HSV-1-GFP (based on strain Patton) on Vero cells [70], VV-GFP (derived on strain western reserve) on HeLa cells [4].

AdV-GFP (based on AdV5) was generated by the Laboratory of Patrick Hearing. The AdV5 E4-ORF3 reading frame was precisely replaced with EGFP in plasmid pTG3602 [71] using PCR, and recombineering in *E*. *coli*, as previously described [72]. To generate infectious virus, the pTG3602-EGFP plasmid was linearized with PacI and 1 **μ**g DNA transfected into 293T cells. Plaques were purified and working virus stocks were generated by passaging virus on 293T cells. The optimum dose for viral assays was determined by limited dilution in A549 cells and high content microscopy for EGFP-positive cells.

Experiments with all above viruses were carried out in biosafety level 2 (BSL2) containment in compliance with institutional and federal guidelines.

The infectious clone of CHIKV La Réunion 06–049 expressing ZsGreen was constructed by the Laboratory of Kenneth Stapleford using standard molecular biology techniques. First, an AvrII restriction enzyme site was inserted 5’ of the subgenomic promoter by site-directed mutagenesis using the primers Forward 5’-CACTAATCAGCTACACCTAGGATGGAGTTCATCCC-3’ and Reverse 5’-GGGATGAACTCCATCCTAGGTGTAGCTGATTAGTG-3’. The CHIKV subgenomic promoter was then amplified by PCR (Forward 5’-CCTAGGCCATGGCCACCTTTGCAAG-3’ and Reverse 5’-ACTAGTTGTAGCTGATTAGTGTTTAG-3’) and subcloned into the AvrII site to generate a CHIKV infectious clone containing two subgenomic promoters. Finally, the ZsGreen cassette was amplified by PCR (Forward 5’-GTGTACCTAGGATGGCCCAGTCCAAGCAC-3’ and Reverse 5’-GCTATCCTAGGTTAACTAGTGGGCAAGGC-3’) from a CHIKV infectious clone obtained from Andres Merits (University of Tartu) and subcloned into the AvrII restriction enzyme site. The complete cassette and subgenomic regions were sequenced to ensure there were no second-site mutations. To generate infectious virus, the plasmid was linearized overnight with NotI, phenol-chloroform extracted, ethanol precipitated, and used for in vitro transcription using the SP6 mMessage mMachine kit (Ambion). In vitro transcribed RNA was phenol-chloroform extracted, ethanol precipitated, aliquoted at 1 mg/ml, and stored at -80°C. 10 **μ**g of RNA was electroporated into BHK-21 cells [73] and virus was harvested 48 h post electroporation. Working virus stocks were generated by passaging virus over BHK-21 cells and viral titers were quantified by plaque assay. Experiments with CHIKV were carried out in biosafety level 3 (BSL3) containment in compliance with institutional and federal guidelines.

### Lentiviral generation and transduction of cells

ISGs with antiviral activity (ELF1, IFITM3, IRF1, BST2) were part of the pSCRPSY lentiviral ISG library and co-expressed tagRFP and a puromycin resistance gene [23]. To generate ELF1 domain deletion and point mutants, ELF1 wild type was amplified using forward primer 5’- ATGGCTGCTGTTGTCCAACAGAAC-3’ and reverse primer 5’- CTAAAAAGAGTTGGGTTCCAGCAGTTC-3’, and cloned into pCR8/GW/TOPO TA (Life Technologies). This entry clone DNA was used as starting point for mutagenesis. Mutation R8A was generated by site directed mutagenesis using Quikchange technology (Agilent), forward primer 5’-TATGAGACCATGGGAGCAGCACTCAGGTACTATTAC-3’ and reverse primer 5’-GTAATAGTACCTGAGTGCTGCTCCCATGGTCTCATA-3’. ELF1 lacking the transcription factor (TF) domain was generated by PCR amplification of N-terminally truncated ELF1, using forward primer 5’-ATGGCTGCTGTTGTCCAACAGAAC-3’ and reverse primer 5’-CTAAAAAGAGTTGGGTTCCAGCAGTTC-3’ and cloning into pCR8/GW/TOPO TA. ELF1 lacking the internal ETS domain was generated using a PCR overlap extension PCR approach. We amplified the N-terminal fragment of ELF1 with forward primer 5’-ATGGCTGCTGTTGTCCAACAGAAC-3’ and reverse primer 5’- GGTGGATTCTAAAGCAGTGTCCAGGGCAAAAGTGGAAGGTCAG-3’, and the C-terminal fragment with forward primer 5’- GCAGTGTCCAGGGCAAAAGTGGAAGGTCAGCGCTTGGTGTATC-3’ and reverse primer 5’-CTAAAAAGAGTTGGGTTCCAGCAGTTC-3’. We then performed overlap extension PCR with the N-terminal and C-terminal PCR products as template, using forward primer 5’-ATGGCTGCTGTTGTCCAACAGAAC-3’ and reverse primer 5’-CTAAAAAGAGTTGGGTTCCAGCAGTTC-3’. The final PCR product was cloned into pCR8/GW/TOPO TA. From pCR8/GW/TOPO TA, ELF1 R8A, dTF and dETS constructs were swapped into pSCRPSY vector by gateway cloning.

To generate lentiviral stocks, we co-transfected 293T Lenti-X cells (Clontech laboratories) with plasmids expressing VSV-G, gag-pol, and the respective pSCRPSY plasmid, at a DNA ratio of 1:5:25. 48–72 h post transfection, we harvested the supernatant, centrifuged to remove cellular debris, and filtered the supernatant through a 0.2 μM filter. We then added HEPES to a final concentration of 20 mM and polybrene to 4 μg/ml. Each lentivirus stock was titrated on the respective cell types and diluted to obtain 90% transduced cells as determined by microscopy.

### ISG induction assays

To determine ISG mRNA expression kinetics, we treated PBMCs or A549 with interferon-beta (Millipore Sigma) at 500 U/ml in the culture medium. At indicated time points, cells were washed once with PBS, then lysed with 375 μl of RLT buffer directly in the culture plate, and total RNA extracted using the RNeasy kit (Qiagen).

For interferon stimulation of human airway epithelial cultures, 500 U/ml of interferon-beta was added to the basolateral chamber. For viral stimulation of human airway epithelial cultures, cultures were washed apically with 50 μl of prewarmed PBS twice for 15 min at 37°C. Virus inoculates (5E6 PFU for influenza A/California/07/2009 (H1N1) virus and 1.12 E7 FFU TCID_50_ for human pararinfluenzavirus 3-EGFP in 50 μl PBS Mg/Ca) were added apically for one hour. Inoculates were then removed and cultures were washed twice with PBS then incubated for the indicated times. For cell lysis, cultures were washed once with PBS, then the membranes were excised from the transwell and transferred into 375 μl of RLT buffer. Cells were homogenized with an RNAse-free plunger, and total RNA extracted from the lysate using the RNeasy kit (Qiagen).

For the in vivo poly(I:C) challenge experiment, C57/BL6 mice were anesthetized with ketamine/xylazine (100mg/10mg/kg), and 100 μg of poly(I:C) (InVivogen), diluted with PBS in a volume of 35 μl, was administered intranasally. For determination of ISG mRNA profiles, animals were sacrificed at indicated time points, whole lungs were collected, homogenized in 500 μl PBS, and debris was spun down at 15000 rpm for 10 min. Total RNA was extracted from the supernatants using Qiashredders and the RNeasy kit (Qiagen).

For in vitro poly(I:C) stimulation, A549 cells in 24-well plates were transfected with 1 μg of poly(I:C) using Lipofectamine LTX reagent, or treated with Lipofectamine LTX alone (carrier control). At indicated time points, cells were washed once with PBS, then lysed with 375 μl of RLT buffer directly in the culture plate, and total RNA extracted using the RNeasy kit (Qiagen).

For gene expression analyses in the absence of endogenous ELF1, we used in vivo morpholino oligomers (MO). A549 cells were supplemented with 50 μM MOs for 24h. Cells were washed once with PBS, then lysed with Trizol and chloroform extracted. Total RNA was extracted using the RNeasy kit (Qiagen). Relative mRNA levels, normalized to housekeeping gene RPS-11, were determined by RT-qPCR (SuperScript III First Strand Synthesis System, Life Technologies and PowerUP SYBR Green Master Mix, Thermo Fisher Scientific). Primer sequences are listed in S10 Table.

### Western blot analyses

Protein levels from cell lysates or mouse lung homogenates were measured by western blotting using anti-ELF1 antibody (1:5000, Santa Cruz for murine Elf1; 1:5000, Bethyl labs for human ELF1), anti-IRF1 antibody (1:1000, Cell Signaling), anti-STAT1-antibody (1:1000, Cell Signaling), anti-STAT3 antibody (1:2000, Cell Signaling), anti-actin antibody (1:1000, Thermo Fisher). Actin was used as housekeeping control using anti-actin-HRP antibody (1:1000, Thermo Fisher) or anti-actin antibody (1:1000, Thermo Fisher). Relative band intensities were determined with ImageJ.

### Viral growth assays by microscopy and image analysis

If not otherwise stated, we used the CellInsight CX7 High-Content Screening (HCS) Platform (Thermofisher) and high-content software (HCS) for microscopy and image analysis. For virus spread experiments, the optimum virus dose and timing of endpoints was determined by high content microscopy prior to experiments for each cell type and each virus. For experiments with the endpoint at one round of viral replication, we chose the time that resulted in bright, yet individual virus-positive cells. For the optimum virus dose, we chose viral dose that yielded reproducible 0.5–3% infected cells at that time point. For the second endpoint at multiple rounds of replication, we chose the time that resulted in 10–60% of infected cells from that viral dose, depending on the spreading capability of the given virus. Experiments with YFV and CHIKV were performed in the presence of 0.4 μM of Ruxolitinib to allow for viral spread on interferon-competent A549 cells.

Experiments analyzing the action of exogenously expressed antiviral ISGs (ELF1, IRF1, IFITM3, BST2) were performed using optimized viral doses and time points. A549 (or A549 STAT1^-/-^) cells in multiple 96-well plates were transduced with pSCRPSY:empty lentivirus for 48h, and then infected with serial dilutions of the respective viruses. For experiments analyzing the specificity of ELF1 knockdown, A549 cells were first transduced to express ELF1 wild type, ELF1 R8A, or empty vector control for 6 h, then media was changed to media containing 15 μM in vivo morpholino oligomers (MO). After 2 d, cells were infected with 100 PFU/well of influenza A/WSN/1933 virus, and infection media was supplemented with 8 μM MOs.

At different times post-infection, each plate was fixed with 1.5% paraformaldehyde for 15 min, washed with PBS, quenched for 5 min with 20 mM NH_4_Cl, and washed with PBS again. To permeabilize the cells, we used 0.1% Triton-X in PBS for 4 min, followed by washing with PBS three times. For reporter viruses expressing a strong GFP-signal (HPIV3, YFV, CHIKV, AdV, HSV-1, CxB and VV), cells were stained with DAPI only. IAV-infected cells were blocked with 1% BSA in PBS for 1 h at room temperature, stained with anti-NP antibody (1:500, BEI resources) for 1 h rocking at 37°C, washed three times with PBS before staining with secondary goat Alexa 488 antibody and DAPI for 1 h rocking at 37°C and finally washed three times with PBS. Plates were imaged using the 4x objective in 9 fields covering the entire 96-well. For this assay, we used the HCS analysis protocol “Target Activation”, and reference levels were set at three standard deviations for the highest background from all mock-infected control wells.

### IAV life cycle assays

We transduced A549 with the indicated lentiviruses, and performed all assays 2d after. For the mini genome assay, transduced cells were transfected, in a 24-well format, with pCAGGS constructs for IAV WSN/33 PB1, PB2, PA (100 ng each), and NP or empty pCAGGS (200ng), as well as the RNA polymerase II-driven Renilla luciferase reporter pRLTK (40 ng), and the IAV-specific RNA polymerase I-driven firefly luciferase reporter (pPolI-luc, 60 ng). Diphyllin or Ribavirin were added as controls 4h before transfection and until the end of the experiment. Cells were harvested 20h post transfection, lysed and assayed using Dual Luciferase Assay Kit (Promega).

For determination of egress efficiency, transduced A549 were challenged with IAV WSN/33 at MOI 0.1. At 24 hpi, cells were harvested for quantitation of intracellular vRNA and supernatants for determination of extracellular vRNA and number of infectious particles. Viral RNA was extracted with RNeasy kit (Qiagen, for cells) or QIAmp Viral RNA kit (Qiagen, for supernatants). For genome quantification by Taqman RT-qPCR we used RealTime ready RNA Virus Master (Roche); primer and probe sequences can be found in S10 Table. The number of infectious particles in the supernatants were determined by plaque assay on MDCK cells using avicel overlay and crystal violet staining (A/WSN/33), or agar overlay followed by NP-immunostaining (A/PR8/1934).

### Cytotoxicity assays

All small molecule inhibitors and morpholinos used in this study were tested for cytotoxicity and optimum effective dose for each cell type. Cells in 96-wells were incubated with a serial dilution of the inhibitor, keeping the carrier concentration constant in each well. Incubation time corresponded to the time the drug would be in contact with the cells in the actual assay. 10% Ethanol was used as positive control for cell death. Cells were then stained with Sytox green (1:20,000, Thermo), washed with DMEM, fixed with 1.5% paraformaldehyde, permeabilized with 0.1% triton X-100, stained with DAPI, and imaged with the 4x objective. For this assay, we used the HCS analysis protocol “Target Activation”, and reference levels were set at three standard deviations from the mean of control wells. Cytotoxicity was evaluated by a reduction of total (DAPI-positive) cells per well, as well as the % of dead (Sytox-positive) cells. Drugs were used at the highest safe dose in the assays described below, i.e. the dose not reducing the number of total cells, and not increasing the number of dead cells as compared to carrier control.

The effect of exogenous ELF1 expression in A549 cells was also tested for cytotoxicity. In a cell outgrowth experiment, A549 cells in a 12-well plate were transduced to express ELF1 wild type, R8A or empty vector control. All constructs coexpressed RFP and puromycin resistance. 48 h post transduction, cells were plated into 6-wells and media containing 10 μg/ml puromycin was added. After 3 days, surviving cells were trypsinized and replated into 96-wells at 800 cells per well. Two plates were prepared, and one plate was fixed with 1.5% PFA once cells became adherent (baseline cell number), and the other was incubated for 3 days, then fixed with 1.5% PFA. To permeabilize the cells, we used 0.1% Triton-X in PBS for 4 min, followed by washing with PBS three times, then stained with DAPI for 1 h rocking at 37°C and finally washed three times with PBS. Number of DAPI-positive cells per well were determined by microscopy as described above.

Cell death was examined by flow cytometry using a Live/Dead staining (Alexa Fluor 350 NHS Ester) according to the manufacturer's instructions. Briefly, 3×10E4 A549 cells were seeded on a 24-well plate. Next day, the cells were transduced with 300 μl of empty, ELF1 or R8A undiluted lentivirus per well. Positive controls for cell death were treated with Staurosporine 1μM for 12 h. All cells were collected 48 h after transduction and stained in 100 μL of FACS buffer using the Live/Dead staining (1:400) at 4°C in the dark for 30 min. Data was acquired using a ZE5 Cell Analyzer and Everest Software (BioRad) and analyzed using FlowJo v.10.6.1 software (Tree Star, Inc., Ashland, OR, USA).

### Luciferase assay for ISRE activity

293T carrying firefly luciferase (Fluc) under the control of a promoter carrying the ISRE motif and stably expressing renilla luciferase (Rluc) as control were plated in 48-well plates and transfected with plasmids encoding GFP as negative control, MDA5 as positive control, or ELF1, ELF2, ELF3, ELF4 and ELF5, respectively. A constant 200 ng of total DNA per well was used, but doses of MDA5 or ELF (transgene) expression plasmids were varied, resulting in the following transfections: 50 ng transgene / 150 ng GFP, 100 ng transgene / 100 ng GFP, and 200 transgene / no GFP. 24 h post DNA transfection, cells were transfected with 100 ng polyI:C / 48-well. After 24 h, cells were harvested, lysed, and assayed for firefly and renilla luciferase signals using the Dual luciferase kit (Promega). Firefly raw data was normalized to the renilla signal.

### IAV low MOI growth kinetics in the presence or absence of ELF1 and controls

To determine IAV growth in the presence of exogenously expressed ELF1, A549 or NHBE in 24-wells were transduced to express ELF1 and controls. 48 h post transduction, cells were gently washed twice with prewarmed PBS, and infected with influenza A/WSN/1933 (H1N1) virus at MOI 0.01 in 200 μl of PBS. The remaining inoculate was stored at -80°C for back-titration. Cells with inoculate were placed in a rocking incubator at 37°C for 1 h, then washed twice with prewarmed PBS, covered with 560μl of prewarmed growth medium, and placed into a regular CO_2_ incubator at 37°C. After 1 h, 50 μl of supernatant were collected and stored at -80°C to determine successful removal of input virus. Supernatant was then collected every 12 h until 48 hpi, and stored at -80°C. During supernatant collections, 50μl of fresh, prewarmed growth medium was replaced in each well to keep total volume constant throughout the 48 hours. Viral titers in the supernatants were determined by plaque assy.

For IAV growth kinetics during knockdown of endogenous ELF1, A549 cells were supplemented with 15μM in vivo morpholino oligomers for 2 d, then infected with influenza A/WSN/1933 virus at MOI 0.01 as described above. During supernatant collections, 50μl of fresh, prewarmed growth medium with 8μM in vivo morpholino oligomers were replaced in each well to keep total volume constant throughout the 48 h. Viral titers in the supernatants were determined by plaque assy.

### IAV plaque assay

IAV infectious titers were determined by plaque assay on MDCK cells. Briefly, MDCK cells in 12-well plates were washed with PBS, and incubated with 1:10 serial dilutions of IAV in PBS for 1 h in a rocking incubator. After 1 h, cells were washed and overlayed with DMEM (Gibco), 1.2% avicel, 0.001% DEAE, 0.45% Sodium Bicarbonate, GlutaMax, non-essential amino acids, penicillin/streptomycin, and 1μg/ml TPCK trypsin. Cells were then placed in a CO_2_ incubator at 37°C for 48 h. To fix cells and visualize plaques, the avicel overlay was aspirated, washed once with PBS, and cells covered with 0.1% crystal violet, 2% ethanol, 20% methanol for 15 min, then washed with water, and plaques counted manually.

### Morpholino-mediated Elf1 knockdown and influenza A virus challenge in vivo

Five-week-old female BALB/c mice were anesthetized by intraperitoneal injection of a mixture of Ketamine and Xylazine (100 μg and 5 μg per gram of body weight), prior to intranasal administration of either PBS or 100 micrograms of peptide-conjugated phosphorodiamidate morpholino oligomers (PPMO) mix (50 micrograms of PPMO1 and 2 each) in 40 μl of PBS, on Day -2 and Day -1. On Day 0, Mice were challenged intranasally with 40 PFU of PR8 IAV (LD50 = 50 PFU) in 40μl PBS. Mice were monitored daily for weight loss and clinical signs. Mice lungs were harvested on Day 3 and Day 6 post infection for measuring viral titers (5 mice per condition). Lung homogenates were prepared using a FastPrep24 system (MP Biomedicals). After addition of 800 μl of PBS containing 0.3% BSA, lungs were subjected to two rounds of mechanical treatment for 10 s each at 6.5 m/s. Tissue debris was removed by low-speed centrifugation, and virus titers in supernatants were determined by plaque assay. A group of mice (5 per condition) were monitored until day 14 post infection for survival.

### RNA-seq and analysis

For ectopic ELF1 RNA-Seq experiments, A549 cells in 24-well plates were transduced with pSCRPSY lentivirus encoding ELF1 wildtype, ELF1 R8A, or empty vector control, or non-transduced. After 48 h in culture, some empty-transduced cells were stimulated with 500 U/ml of interferon-beta (Millipore). 42 h later, other empty-transduced cells were stimulated with 500 U/ml of interferon-beta (Millipore). Six hours later all cells were harvested (96 h post transduction, and 6 or 48 h post IFN-treatment, see Fig 6A) as follows: medium was aspirated, cells were washed with PBS, lysed in RLT buffer, and RNA extracted following the RNeasy kit protocol (Qiagen). The experiment was performed three times, with each transduction condition represented once in each replicate "batch." RNA-Seq libraries (for all samples in a single batch) were prepared with the Illumina TruSeq Stranded Total RNA Library Prep Kit according to manufacturer's instructions, and sequenced on the Illumina NextSeq 500 platform at 75nt read length in single-end configuration. One ELF1-R8A replicate (batch 1) failed library preparation and was not included in sequencing or analysis.

Reads were mapped to the human genome reference (hg19) supplemented with the pSCRPSY plasmid sequence (containing EGFP gene), using the HISAT2 (v2.1.0) alignment tool [74] with Ensembl v75 gene annotations (supplemented with pSCRPSY gene annotation) and the "—rna-strandness R" and "—dta" parameters. Read counts per gene were quantified against Ensembl (v75) transcript reference annotations (appended with gene annotation for pSCRPSY, "MSTRG.1") using featureCounts (v1.6.3)[75]. All further analyses were conducted using R (v3.5.0). Genes with greater than or equal to 3 read counts in at least 3 samples were defined as “expressed” and included in downstream analyses. For principal component analysis (PCA), read counts were normalized and variance stabilized by regularized log transformation (rlog function, DESeq2 package v1.20.1 [76]). Replicate batch effects were corrected with the removeBatchEffect function in the limma package (v3.36.1). PCA was conducted on the 1000 most variable genes across all samples.

For differential gene expression analysis, raw read counts were TMM-normalized and log_2_ transformed with voom (limma v3.36.1) [77,78]. Differential gene expression testing was performed with a linear model incorporating a factor for experimental condition. Replicate batch effects were estimated with the duplicateCorrelation function and included in the model. To define the ELF1 transcriptional program, pairwise tests were conducted for ELF1 (WT) vs (ELF1 R8A), and ELF1 (WT) vs empty vector contrasts. Differential gene expression test p values were adjusted for multiple testing by the method of Benjamini and Hochberg [79]. In order to focus further analyses on those genes markedly affected by ELF1, a relatively stringent filter was applied to differential expression results: “ELF1 differentially expressed genes” were defined as those genes with adjusted p value < 0.05 and log_2_ fold-change ≥ 2 (or ≤ -2) in both ELF1 (WT) vs (ELF1 R8A), and ELF1 (WT) vs Empty vector contrasts. GO term enrichment analysis in ELF1 differentially expressed genes was performed with the GOSeq tool (v1.32.0) [80], and results visualized with GOplot (v1.0.2)[81]. To identify ISGs, pairwise tests were conducted for interferon 6 h (empty vector) vs mock (empty vector), and interferon 48 h (empty vector) vs mock (empty vector) contrasts. Statistical thresholds were applied as for above ELF1 contrasts. To statistically evaluate differences in the ELF1 transcriptional program and interferon response, additional differential expression testing was performed for (ELF1-WT vs ELF-R8A) vs (interferon 6 h vs mock) and (ELF1-WT vs ELF-R8A) vs (interferon 48 h vs mock) contrasts with the same thresholds.

### ChIP-seq and analysis

To cross-link proteins to the DNA, A549 cells were treated with 1% formaldehyde for 10 minutes and the cross-linking reaction was terminated by addition of glycine to a final concentration of 0.125 M. Cross-linked chromatin was sonicated using a Biorupter (Diagenode) to generate DNA-fragments of approximately 200 to 600 bp in length. The optimum sonication time for A549 cells was 8 min. Anti-ELF1 antibody (Santa Cruz) was pre-bound to Invitrogen Dynal magnetic beads (Invitrogen Dynabeads anti-rabbit M-280) in 0.5% BSA/PBS. ChIP-Seq was performed using 1.25x10^7^ cells and 0.5μg antibody coupled to 35μL magnetic beads. Beads were added to an 800μl volume of cell lysates. After overnight incubation, beads were washed 8x in modified RIPA wash buffer (50 mM HEPES [pH 7.6], 100 mM LiCl, 1mM EDTA, 1% NP-40 and 0.7% Na-deoxycholate) and 1x in TE containing 50 mM NaCl. Elution of DNA was performed in TE buffer containing 1% SDS. After overnight cross-link reversal at 65°C, RNase digestion and Proteinase K digestion, ChIP DNA and input DNA were purified using the QIAGEN Qiaquick PCR purification kit. For ChIP-Seq library generation, 34μL of ChIP DNA was used to generate blunt-ended DNA using reagents supplied with the End-It DNA END-Repair kit (Lucigen). The end-repaired DNA was purified using the QIAGEN Qiaquick PCR purification kit. Using Klenow Fragment (NEB) “A” bases were added to the DNA. DNA was purified using the QIAGEN MinElute kit. T4 DNA ligase (NEB) was used for ligation of Illumina/Solexa PE adapters to the DNA fragments. The adaptor-ligated DNA was purified with the QIAGEN MinElute kit. The DNA fragments were subjected to 18 cycles of PCR using the Illumina/Solexa primers PE 1.0 and 2.0 to generate the ChIP-Seq libraries. The ChIP-Seq libraries were then purified with the QIAGEN MinElute kit.

Libraries from two biological replicates of ELF1 ChIP and two biological replicates of Input control were subject to 50-bp single end sequencing on the Illumina HiSeq 2500 platform. Reads were aligned to the hg19 build of the human genome using bowtie2 version 2.3.4.1 using default parameters. Reads with a quality score below 30 and duplicate mapped reads were removed using samtools version 1.9 and picard-tools version 1.88, respectively. After confirming that read coverages between biological replicates correlated well with each other (pearson correlation coefficient > 0.9), respective BAM alignment files for two input controls and two ELF1 ChIP replicates were merged (S10 Fig). The merged input and ELF1 ChIP BAM files were then converted to BED files and mitochondrial reads were removed using bedtools version 2.27.1 bamToBed function with default parameters. Mapped reads were then extended by 250 bp (average length of library fragments) using bedtools slop function. Coverage was calculated using bedtools genomecov function with a per-million scaling factor (calculated as 1000000 / total number of mapped reads) to normalize for library size. hg19 chromosome size metrics for coverage calculations were obtained from UCSC: https://genome.ucsc.edu/goldenpath/help/hg19.chrom.sizes). The resulting bedGraph files were converted to a bigwig file using ucscutils version 374 bedGraphToBigWig function. Bigwig files were viewed using Integrative Genomics Viewer 2.4.16 and Gviz version 1.28.1. Peaks were called using MACS 2.1.1 with a stringent q-value cut off of 0.0001, resulting in a total of 12,464 peaks. Peaks and were annotated to nearby genes using homer version 4.10, resulting in 9,515 unique genes being associated with a peak. Annotation with homer version 4.10 also gave location of peak relative to associated gene (i.e. promoter, 5’ UTR, intron, exon, 3’ UTR, transcription termination site, intergenic region etc.).

Metagene analysis was done using deeptools version 3.1.0. First, log_2_(reads per million mapped reads of ELF1 ChIP / reads per million mapped reads of input control) ChIP enrichment values were calculated across 100 bp windows for the entire human genome. Then, all refseq gene transcription start and end sites were obtained from UCSC (hgdownload.soe.ucsc.edu/goldenPath/hg19/database/refGene.txt.gz). The GTF file format was converted to BED file format after separating genes by strand location. Finally, mean log_2_ ChIP enrichment values were plotted for 10 bp bins along all refseq gene bodies scaled to a length of 5kb, plus 3kb upstream and downstream regions after transcription start and end sites.

### Quantification and statistical analysis

All n of in vitro experiments are from biologically independent experiments. Statistical analysis was performed in Prism (GraphPad Software, Version 7.0e, 2018). The statistical tests used and the number of biological replicates is indicated in each figure legend. Unless otherwise stated two conditions were compared using two-tailed Student’s *t*-tests. Statistical significance was defined as a p value of 0.05.

## Supporting information

S1 Fig Related to Fig 1. **a.** Primary normal human bronchial epithelial cells (NHBE), **b.** 293T cells, or **c.** HeLa cells were treated with interferon-beta. Western blots show ELF1 or IRF1 levels at 0 or 6 h post stimulation. **d.** A549 cells were transfected with polyI:C or carrier control. ELF1 or RSAD2 (viperin; ISG control) mRNA levels were determined by RT-qPCR and normalized relative to housekeeping gene RPS-11. Fold increase over pre-treatment control levels from n = 3 replicates. RT-qPCR data shown as individual replicates; line represents the mean. Paired t-test compared to carrier, ***p<0.001. **e.** C57BL/6 mice were intranasally challenged with polyI:C, sacrificed at the indicated time points and mRNA levels of Elf1 or Ifitm3 (control ISG) determined by RT-qPCR from lung homogenates. n = 5 mice per time point. Paired t-test compared to 0 h time point, **p<0.01.
(TIF)

S2 Fig Related to Fig 2. **a,b.** A549 were transduced to express transgenes and RFP as control, and challenged with influenza A/WSN/1933 virus (IAV). **a.** Mean ± SEM of % RFP-positive (transduced) cells by high content microscopy, corresponding to experiments in Fig 2B. Transduction efficiency at 12 h post IAV infection (left y-axis) or 48 h post IAV infection (right y-axis). **b.** 48 h post transduction, cells were challenged with a high MOI of IAV, and % of virus-infected (NP-positive) cells determined by high content microscopy after one replication cycle (8 hpi). Mean ± SEM of % IAV-infected cells by high content microscopy in A549 expressing ELF1 wild type (WT) or loss-of-function mutant (R8A), IFITM3 as early (entry) ISG inhibitor control, or empty vector as negative control (n = 3). **c.** Schematic of MO-mediated knockdown and transgene rescue in A549 expressing ELF1 wild type, R8A, or empty negative control. **d.** Mean ± SEM of % influenza A/WSN/1933 virus-infected (NP-positive) cells by microscopy, n = 3. t-test comparing matching NTC and ELF1-knockdown samples, **p<0.01.
(TIF)

S3 Fig Related to Fig 2. **Influenza A virus life cycle assays. a-e**. A549 cells were transduced to express the indicated ISGs. Empty vector served as negative control, and the following positive controls were used for individual IAV life cycle steps: Diphyllin for IAV entry, Ribavirin for IAV replication, Oseltamivir for IAV budding and detachment, IFITM3 for IAV entry, BST2 for IAV egress. Data are represented as mean ± SEM from at least n = 3 independent experiments for all panels. **a.** A549 were challenged with influenza A/WSN/33 virus at MOI 1, and the number of NP-positive nuclei was determined by microscopy at 6 hpi. One-way ANOVA and Dunn’s multiple comparison test. *p<0.1, **p<0.01, ***p<0.001. **b.** IAV replication efficiency was assayed by a luciferase-based IAV minigenome assay in 293T cells. Expression constructs for components of the IAV replication machinery (PB1, PB2, PA and NP, of A/WSN/1933 origin) were co-transfected with a reporter construct mimicking the viral genome, leading to expression of firefly luciferase when the genome mimic is replicated. Individual t-tests compared to empty control, ***p<0.001. **c.** Influenza A/PR/8/1934-NS1-GFP virus single cycle replication was assayed by flow cytometry, determining the percentage of infected (GFP-positive) A549 at 10 hpi, in the ISG-expressing (RFP-positive) population. Individual t-tests compared to empty control, **p<0.01, ***p<0.001. **d.+e.** A549 were infected with influenza A/WSN/1933 virus at MOI 1, washed, and assayed at 12 hpi. d. viral RNA (vRNA) was extracted from supernatants, and vRNA copy number was determined by RT-qPCR. **e.** Infectious virus titers in the supernatant were determined by plaque assay on MDCK cells. Individual t-tests compared to empty control, *p<0.1, **p<0.01, ***p<0.001.
(TIF)

S4 Fig Related to Fig 4. **Transduction efficiencies for assays in Fig 4E-l.** A549 were transduced to express ELF1 or controls. 48 h post transduction, cells were challenged with a low MOI of the indicated viruses and % of infected cells determined by high content microscopy at the late endpoint (endpoint of experiment). Transduction efficiency shown as mean +/- SEM of % RFP-positive (transduced) cells for assay: **a.** ELF mutant analysis with influenzaA/WSN/1933 (H1N1), **b.** influenza A/WSN/1933 (H1N1), **c.** human parainfluenzavirus 3-EGFP, **d.** yellow fever virus-Venus, **e.** chikungunya-virus-ZsGreen, **f.** coxsackievirus-EGFP, **g.** adenovirus-EGFP, **h.** herpes simplex virus 1-EGFP, or **i.** vaccinia virus-EGFP.
(TIF)

S5 Fig Related to Fig 4. **Representative images of late time points for assays in Fig 6.** A549 were transduced to express empty vector as negative control or ELF1 wild type. 48 h post transduction, cells were challenged with a low MOI of the indicated viruses and imaged by high content microscopy at indicated time points post infection. Representative composite images (red cells, transduced; green cells, infected; yellow cells, double-positive) at multi-cycle replication for the following viruses: **a.** influenza A/WSN/1933 (H1N1), stained for NP, **b.** human parainfluenzavirus 3-EGFP, **c.** yellow fever virus-Venus, **d.** chikungunyavirus-ZsGreen, **e.** coxsackievirus-EGFP, **f.** adenovirus-EGFP, **g.** herpes simplex virus 1-EGFP, or **h.** vaccinia virus-EGFP.
(TIF)

S6 Fig Related to Fig 4. **Cytotoxicity assays for ectopic ELF1 and control gene expression on A549. a.** A549 were transduced to express empty vector as negative control, ISG and transcription factor IRF1 as positive control, ELF1 wild type, or ELF1 R8A, a DNA binding domain mutant. Assay for cell growth inhibition. Transduced cells were selected with puromycin for 3 days, then re-plated into two plates at 600 cells/96-well. One plate was fixed at 6 h post plating (once cells adhered), the other plate 3 days later. Cells were stained with DAPI and counted by microscopy. **b.** Retrospective analysis of cell numbers at endpoint of viral challenge assays. Cells numbers from eight independent assays in Fig 4 were determined by microscopy at the endpoint of experiments. **c,d.** Cell death assay by flow cytometry. Transduced A549 were detached, incubated with Alexa350-labeled cell death stain, and number of stained (dead) cells determined by flow cytometry. Staurosporine served as positive control to induce cell death. Representative flow cytometry plots are shown in (**c**), and mean +/- SEM of n = 4 biological replicates summarized in (**d**).
(TIF)

S7 Fig Related to Fig 4A–4C. A549 cells were treated with indicated amounts of the pan-Jak inhibitor Ruxolitinib (Rux), or DMSO carrier control, and infected with YFV-Venus. Cells were imaged and cell numbers or YFV-Venus positive cells determined by microscopy. A. Cell count per well (DAPI-positive) 72 post Rux treatment. **b.** % YFV-Venus positive cells. **c.** Representative images of (**a**) and (**b**). **d**. Analysis of STAT3 phosphorylation as a readout of Jak activity. A549 cells were treated with 500 U/ml of interferon-beta, indicated amounts of Rux, or DMSO carrier control. At 48h post treatment, cells were harvested and analyzed by western blotting using anti-pSTAT3 antibody, or anti-actin antibody as loading control.
(TIF)

S8 Fig Related to Fig 5C. A549 STAT1^-/-^ cells were transduced to express ELF1 or controls. 48 h post transduction, cells were challenged with a low MOI of influenza A/WSN/1933 virus. Mean +/- SEM of % RFP-positive (transduced) cells was determined by high content microscopy at 36 hpi (endpoint of experiment).
(TIF)

S9 Fig Related to Fig 6. **a.** RNA-Seq read counts per million (not normalized) from Fig 6 (**b**) for type I, II and III interferons and additional select cytokines (Ensembl v75). **b.** “Bubble plot” depicting GO terms enriched in ELF1 differentially expressed genes. Each bubble represents a significant (GOSeq adjusted p-value < 0.05) GO term. y-axis indicates enrichment significance (-log_10_ adjusted p-value) and x-axis indicates gene expression fold-change score ([upregulated genes–downregulated genes]/ √number of genes]) for term member genes. Bubble size is proportional to the number of term member genes. GO categories (Biological Process, Cellular Component, Molecular Function) are presented as separate panels to facilitate visualization. Highly significant enriched GO terms (adjusted p-value < 10^−3^) are annotated.
(TIF)

S10 Fig Corresponding to Fig 7. Pearson correlation of ELF1 ChIP and Input replicates. Read coverages were calculated for aligned reads from two ELF1 ChIP biological replicates and two Input control biological replicates with a bin size of 1kb and step size of 100 bp. Shown is a heatmap of pairwise Pearson correlation values of read coverages between the different samples.
(TIF)

S1 Table RNA-seq 381 ELF1-WT unique genes, related to Fig 6. (XLSX)

S2 Table RNA-seq interferon 6 h vs empty, related to Fig 6. (XLSX)

S3 Table RNA-seq interferon 48 h vs empty, related to Fig 6. (XLSX)

S4 Table RNA-seq interferon 6 h vs ELF1-WT, related to Fig 6. (XLSX)

S5 Table RNA-seq interferon 48 h vs ELF1-WT, related to Fig 6. (XLSX)

S6 Table Gene Ontology RNA-seq ELF1 unique all categories, related to Fig 6. (XLSX)

S7 Table ChIP-seq Annotation of peaks, related to Fig 7. (XLSX)

S8 Table ChIP-seq Intersect of ELF1 peaks and ELF1 DEG, related to Fig 7. (XLSX)

S9 Table Material and sources, related to Methods. (XLSX)

S10 Table Oligonucleotides, related to Methods. (XLSX)
