# Supplementary figures and images for: The ETS transcription factor ELF1 regulates a broadly antiviral program distinct from the type I interferon response
Source: PLoS Pathog. 2019 Nov 4;15(11):e1007634. doi: 10.1371/journal.ppat.1007634 (PMC6932815; doi:10.1371/journal.ppat.1007634)

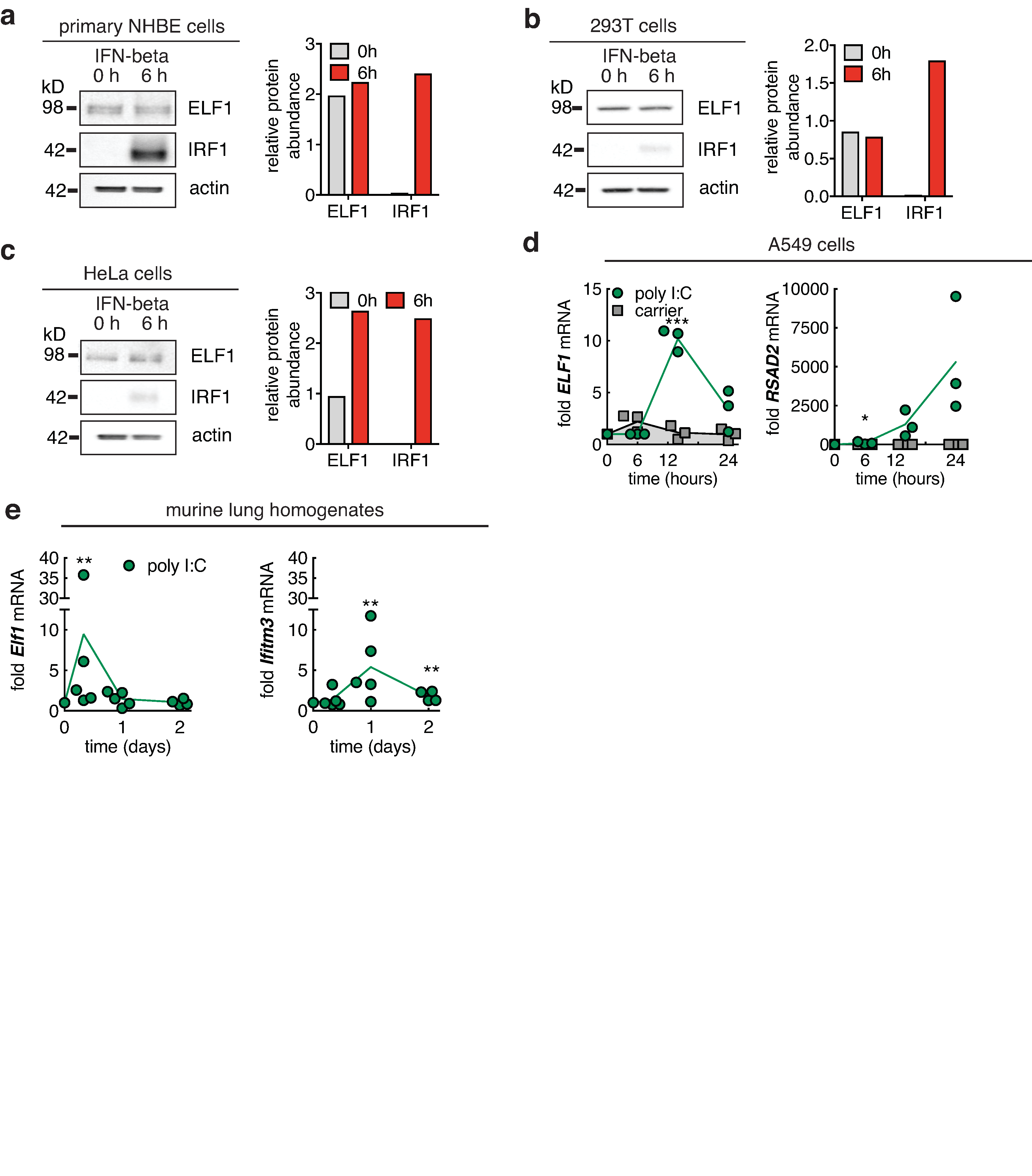

Supplement: S1 Fig — a. Primary normal human bronchial epithelial cells (NHBE), b. 293T cells, or c. HeLa cells were treated with interferon-beta. Western blots show ELF1 or IRF1 levels at 0 or 6 h post stimulation. d. A549 cells were transfected with polyI:C or carrier control. ELF1 or RSAD2 (viperin; ISG control) mRNA levels were determined by RT-qPCR and normalized relative to housekeeping gene RPS-11. Fold increase over pre-treatment control levels from n = 3 replicates. RT-qPCR data shown as individual replicates; line represents the mean. Paired t-test compared to carrier, ***p<0.001. e. C57BL/6 mice were intranasally challenged with polyI:C, sacrificed at the indicated time points and mRNA levels of Elf1 or Ifitm3 (control ISG) determined by RT-qPCR from lung homogenates. n = 5 mice per time point. Paired t-test compared to 0 h time point, **p<0.01. (TIF) [file ppat.1007634.s001.tif]

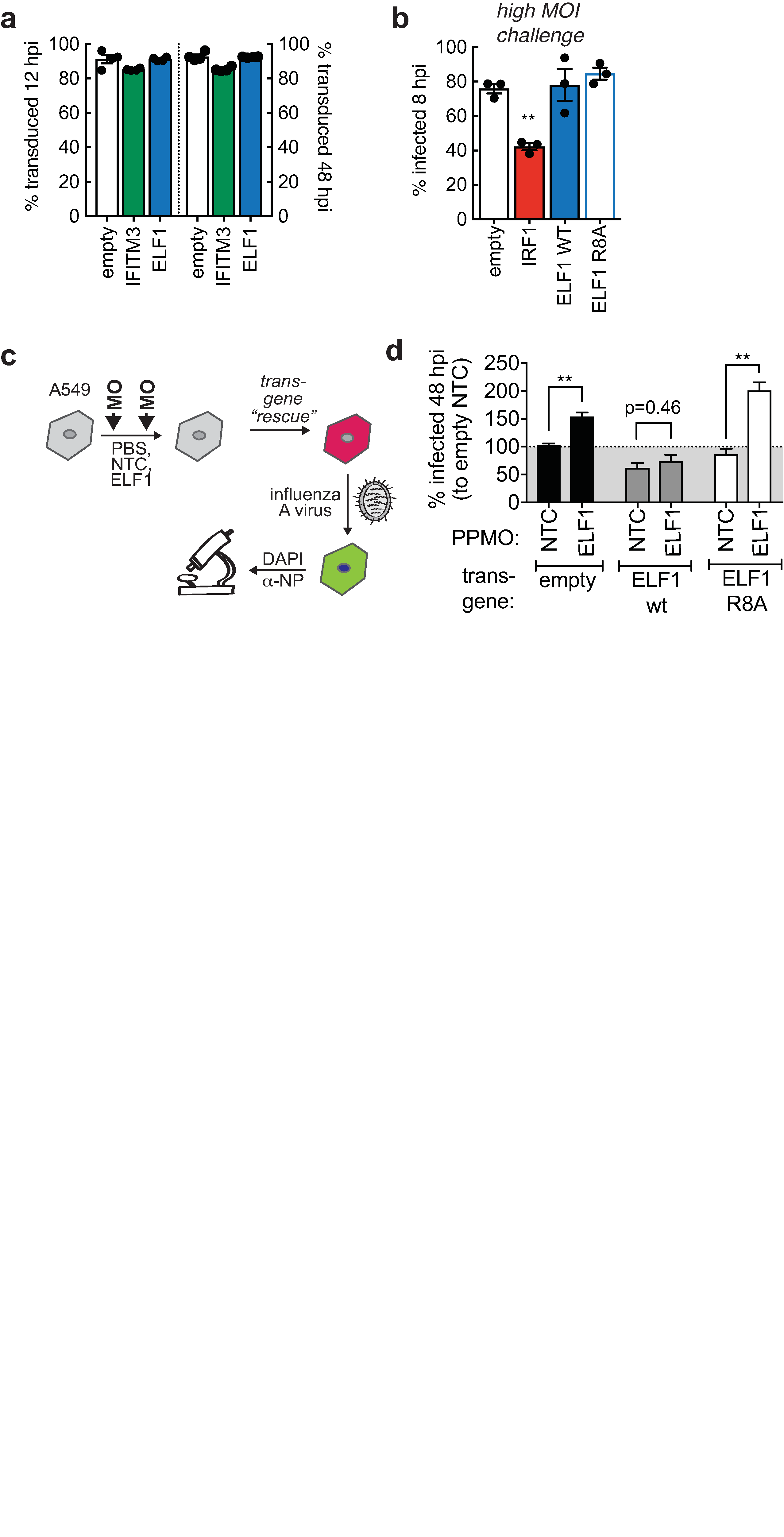

Supplement: S2 Fig — a,b. A549 were transduced to express transgenes and RFP as control, and challenged with influenza A/WSN/1933 virus (IAV). a. Mean ± SEM of % RFP-positive (transduced) cells by high content microscopy, corresponding to experiments in Fig 2B. Transduction efficiency at 12 h post IAV infection (left y-axis) or 48 h post IAV infection (right y-axis). b. 48 h post transduction, cells were challenged with a high MOI of IAV, and % of virus-infected (NP-positive) cells determined by high content microscopy after one replication cycle (8 hpi). Mean ± SEM of % IAV-infected cells by high content microscopy in A549 expressing ELF1 wild type (WT) or loss-of-function mutant (R8A), IFITM3 as early (entry) ISG inhibitor control, or empty vector as negative control (n = 3). c. Schematic of MO-mediated knockdown and transgene rescue in A549 expressing ELF1 wild type, R8A, or empty negative control. d. Mean ± SEM of % influenza A/WSN/1933 virus-infected (NP-positive) cells by microscopy, n = 3. t-test comparing matching NTC and ELF1-knockdown samples, **p<0.01. (TIF) [file ppat.1007634.s002.tif]

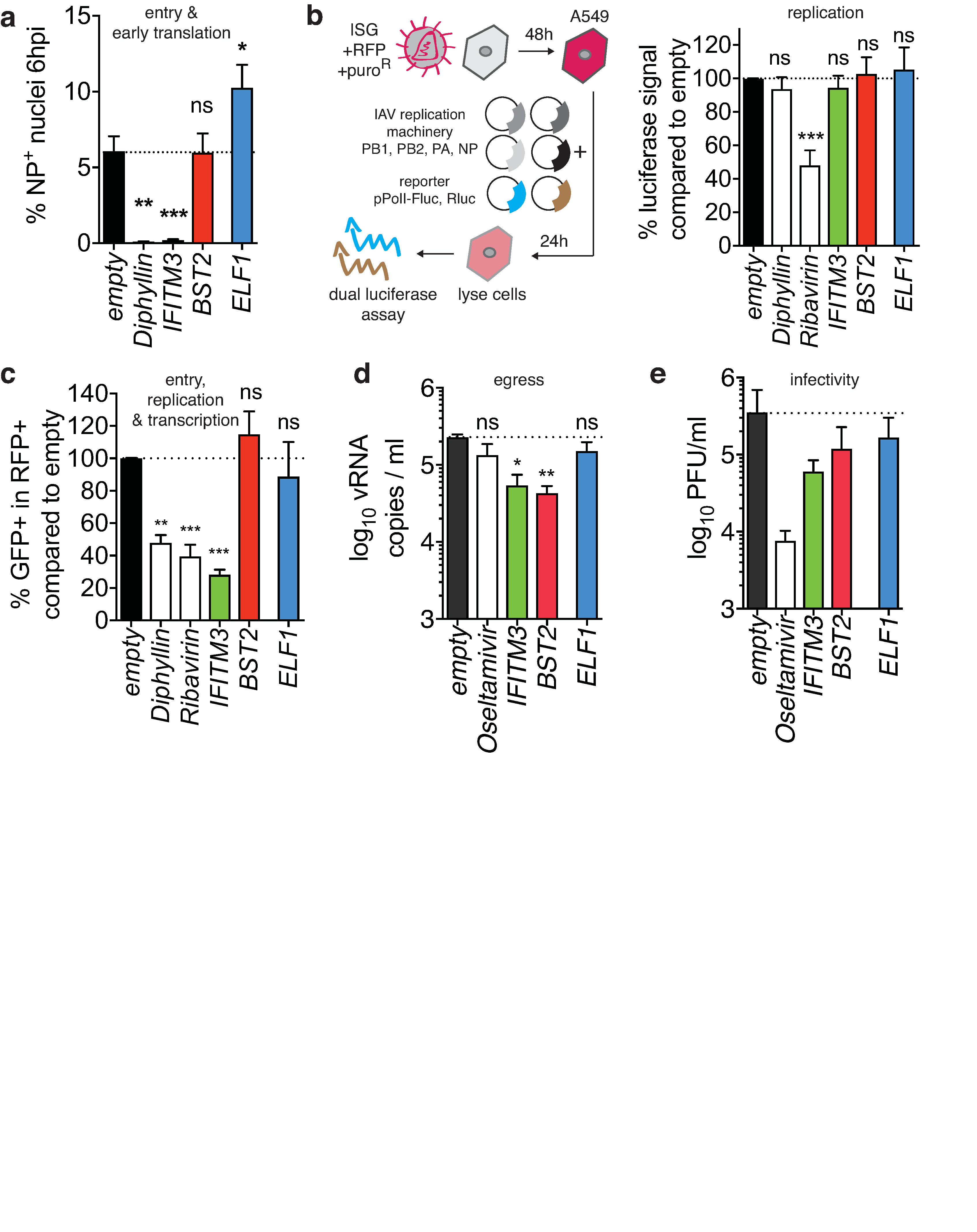

Supplement: S3 Fig — Influenza A virus life cycle assays. a-e. A549 cells were transduced to express the indicated ISGs. Empty vector served as negative control, and the following positive controls were used for individual IAV life cycle steps: Diphyllin for IAV entry, Ribavirin for IAV replication, Oseltamivir for IAV budding and detachment, IFITM3 for IAV entry, BST2 for IAV egress. Data are represented as mean ± SEM from at least n = 3 independent experiments for all panels. a. A549 were challenged with influenza A/WSN/33 virus at MOI 1, and the number of NP-positive nuclei was determined by microscopy at 6 hpi. One-way ANOVA and Dunn’s multiple comparison test. *p<0.1, **p<0.01, ***p<0.001. b. IAV replication efficiency was assayed by a luciferase-based IAV minigenome assay in 293T cells. Expression constructs for components of the IAV replication machinery (PB1, PB2, PA and NP, of A/WSN/1933 origin) were co-transfected with a reporter construct mimicking the viral genome, leading to expression of firefly luciferase when the genome mimic is replicated. Individual t-tests compared to empty control, ***p<0.001. c. Influenza A/PR/8/1934-NS1-GFP virus single cycle replication was assayed by flow cytometry, determining the percentage of infected (GFP-positive) A549 at 10 hpi, in the ISG-expressing (RFP-positive) population. Individual t-tests compared to empty control, **p<0.01, ***p<0.001. d.+e. A549 were infected with influenza A/WSN/1933 virus at MOI 1, washed, and assayed at 12 hpi. d. viral RNA (vRNA) was extracted from supernatants, and vRNA copy number was determined by RT-qPCR. e. Infectious virus titers in the supernatant were determined by plaque assay on MDCK cells. Individual t-tests compared to empty control, *p<0.1, **p<0.01, ***p<0.001. (TIF) [file ppat.1007634.s003.tif]

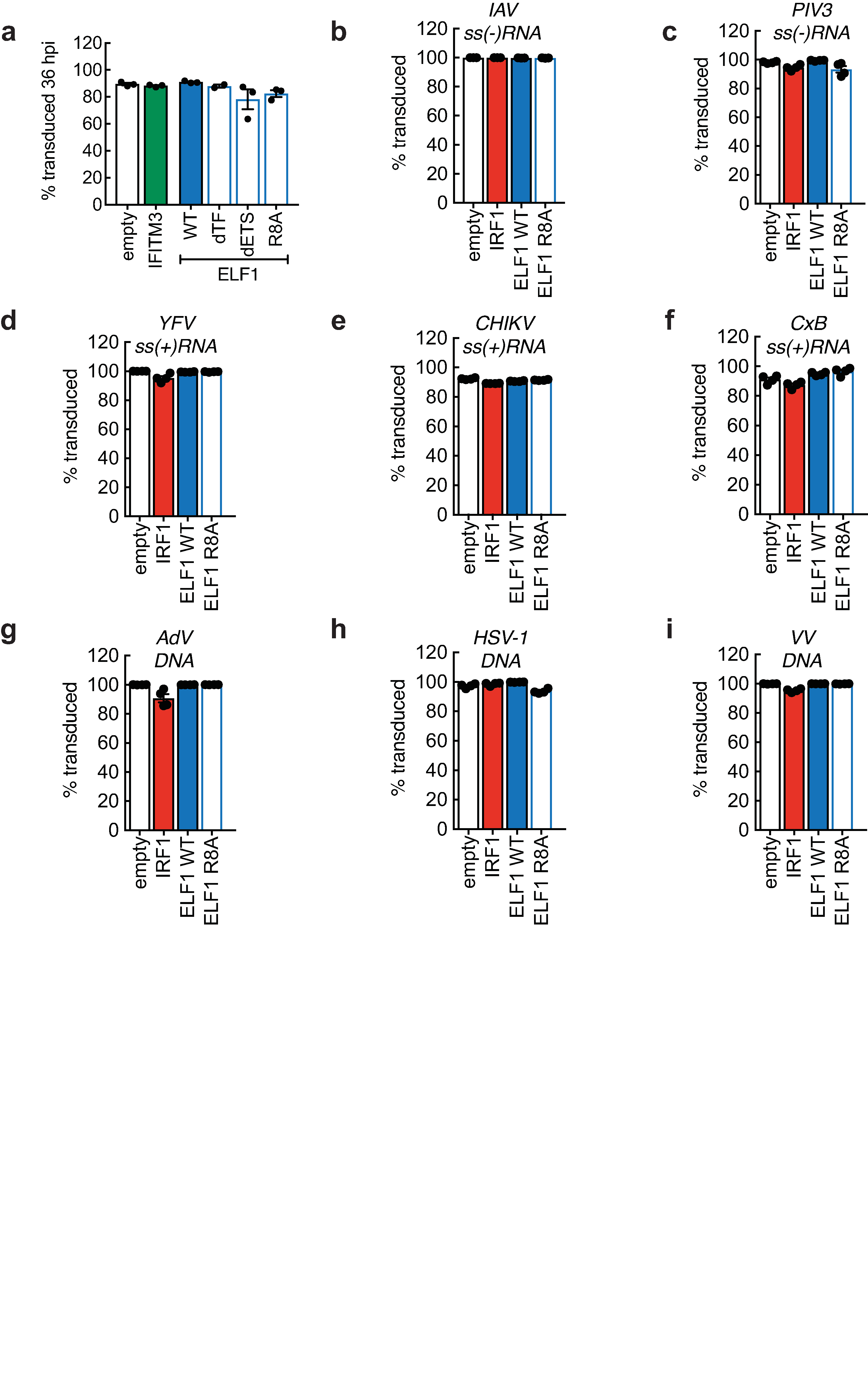

Supplement: S4 Fig — Transduction efficiencies for assays in Fig 4E-l. A549 were transduced to express ELF1 or controls. 48 h post transduction, cells were challenged with a low MOI of the indicated viruses and % of infected cells determined by high content microscopy at the late endpoint (endpoint of experiment). Transduction efficiency shown as mean +/- SEM of % RFP-positive (transduced) cells for assay: a. ELF mutant analysis with influenzaA/WSN/1933 (H1N1), b. influenza A/WSN/1933 (H1N1), c. human parainfluenzavirus 3-EGFP, d. yellow fever virus-Venus, e. chikungunya-virus-ZsGreen, f. coxsackievirus-EGFP, g. adenovirus-EGFP, h. herpes simplex virus 1-EGFP, or i. vaccinia virus-EGFP. (TIF) [file ppat.1007634.s004.tif]

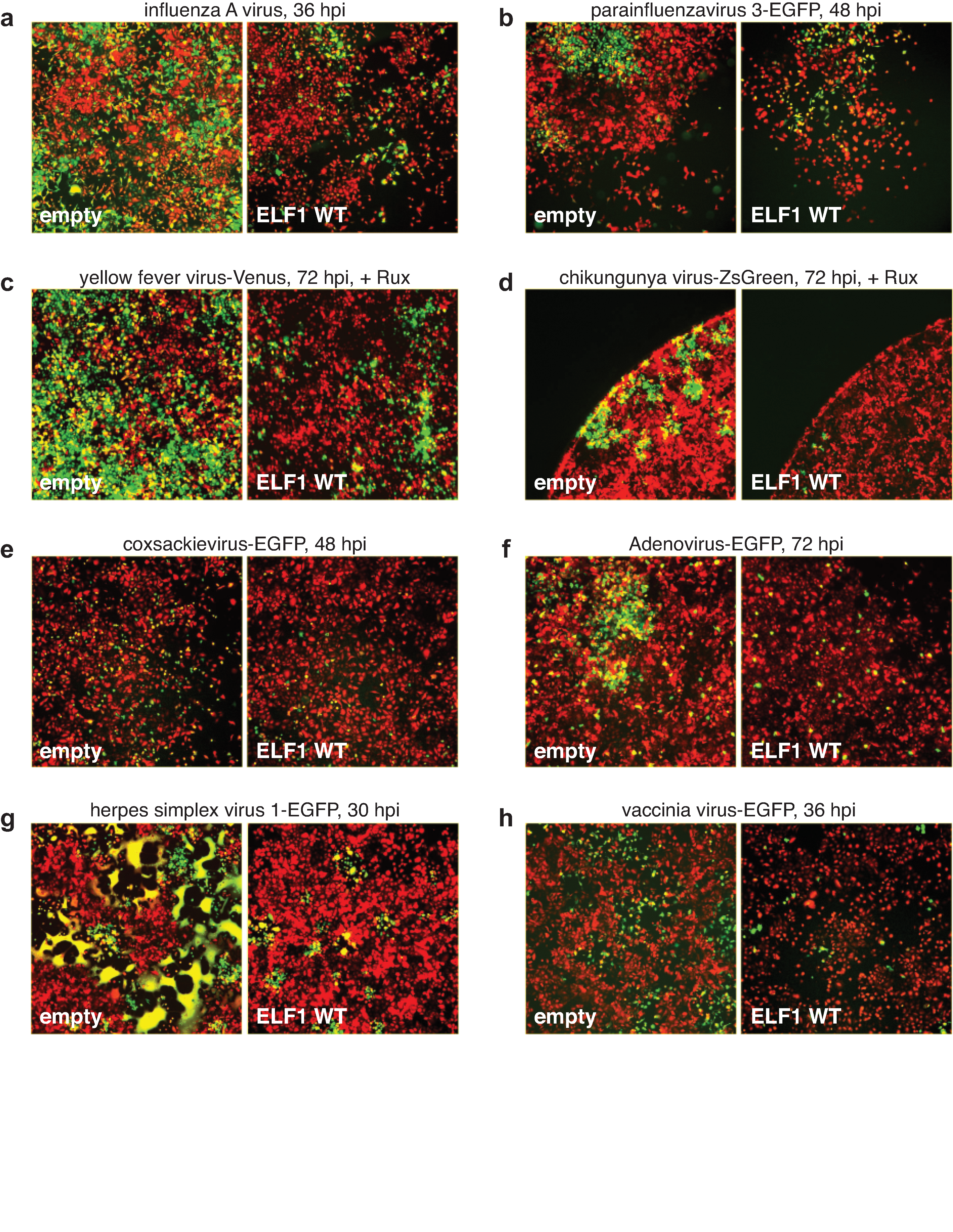

Supplement: S5 Fig — Representative images of late time points for assays in Fig 6. A549 were transduced to express empty vector as negative control or ELF1 wild type. 48 h post transduction, cells were challenged with a low MOI of the indicated viruses and imaged by high content microscopy at indicated time points post infection. Representative composite images (red cells, transduced; green cells, infected; yellow cells, double-positive) at multi-cycle replication for the following viruses: a. influenza A/WSN/1933 (H1N1), stained for NP, b. human parainfluenzavirus 3-EGFP, c. yellow fever virus-Venus, d. chikungunyavirus-ZsGreen, e. coxsackievirus-EGFP, f. adenovirus-EGFP, g. herpes simplex virus 1-EGFP, or h. vaccinia virus-EGFP. (TIF) [file ppat.1007634.s005.tif]

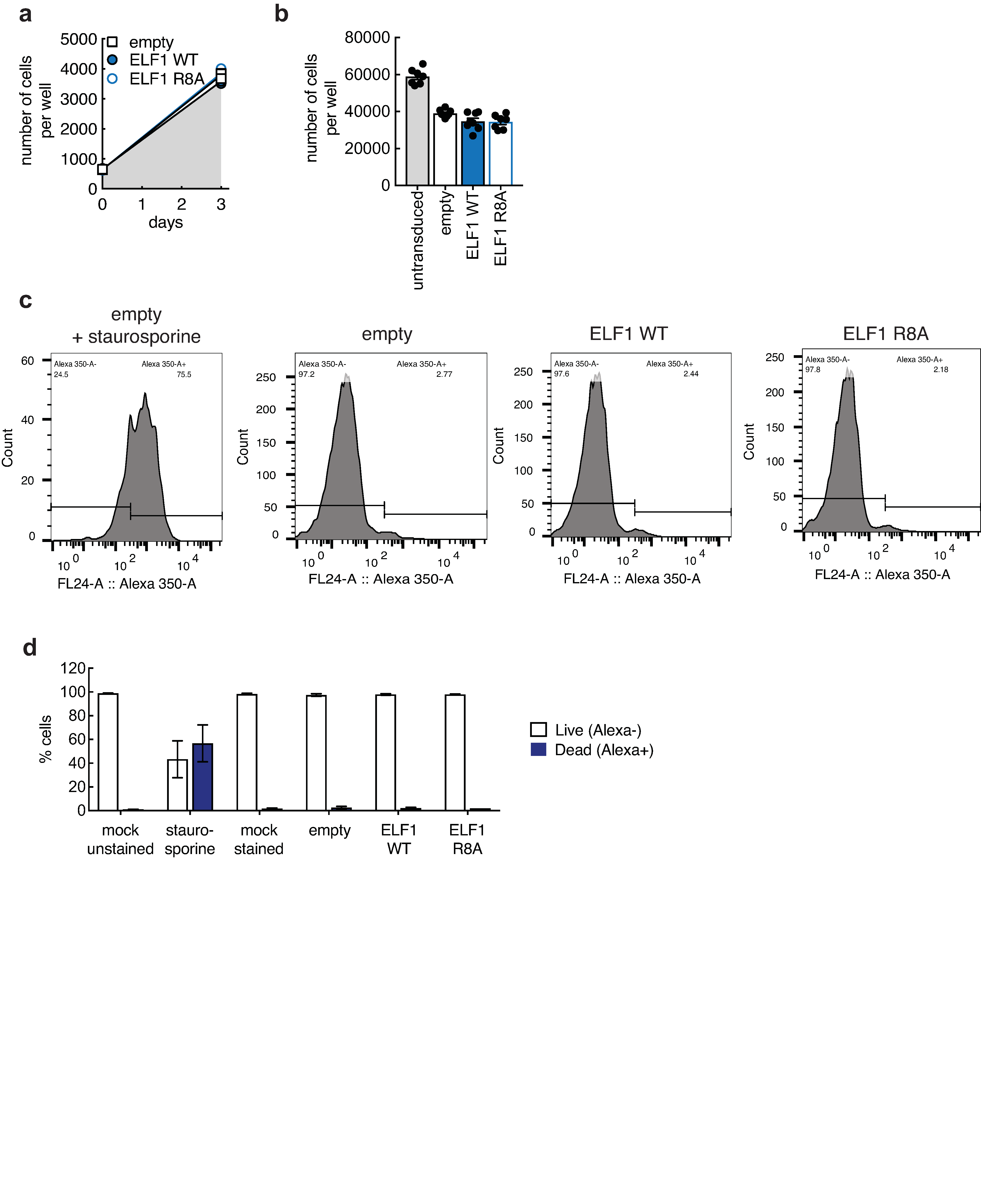

Supplement: S6 Fig — Cytotoxicity assays for ectopic ELF1 and control gene expression on A549. a. A549 were transduced to express empty vector as negative control, ISG and transcription factor IRF1 as positive control, ELF1 wild type, or ELF1 R8A, a DNA binding domain mutant. Assay for cell growth inhibition. Transduced cells were selected with puromycin for 3 days, then re-plated into two plates at 600 cells/96-well. One plate was fixed at 6 h post plating (once cells adhered), the other plate 3 days later. Cells were stained with DAPI and counted by microscopy. b. Retrospective analysis of cell numbers at endpoint of viral challenge assays. Cells numbers from eight independent assays in Fig 4 were determined by microscopy at the endpoint of experiments. c,d. Cell death assay by flow cytometry. Transduced A549 were detached, incubated with Alexa350-labeled cell death stain, and number of stained (dead) cells determined by flow cytometry. Staurosporine served as positive control to induce cell death. Representative flow cytometry plots are shown in (c), and mean +/- SEM of n = 4 biological replicates summarized in (d). (TIF) [file ppat.1007634.s006.tif]

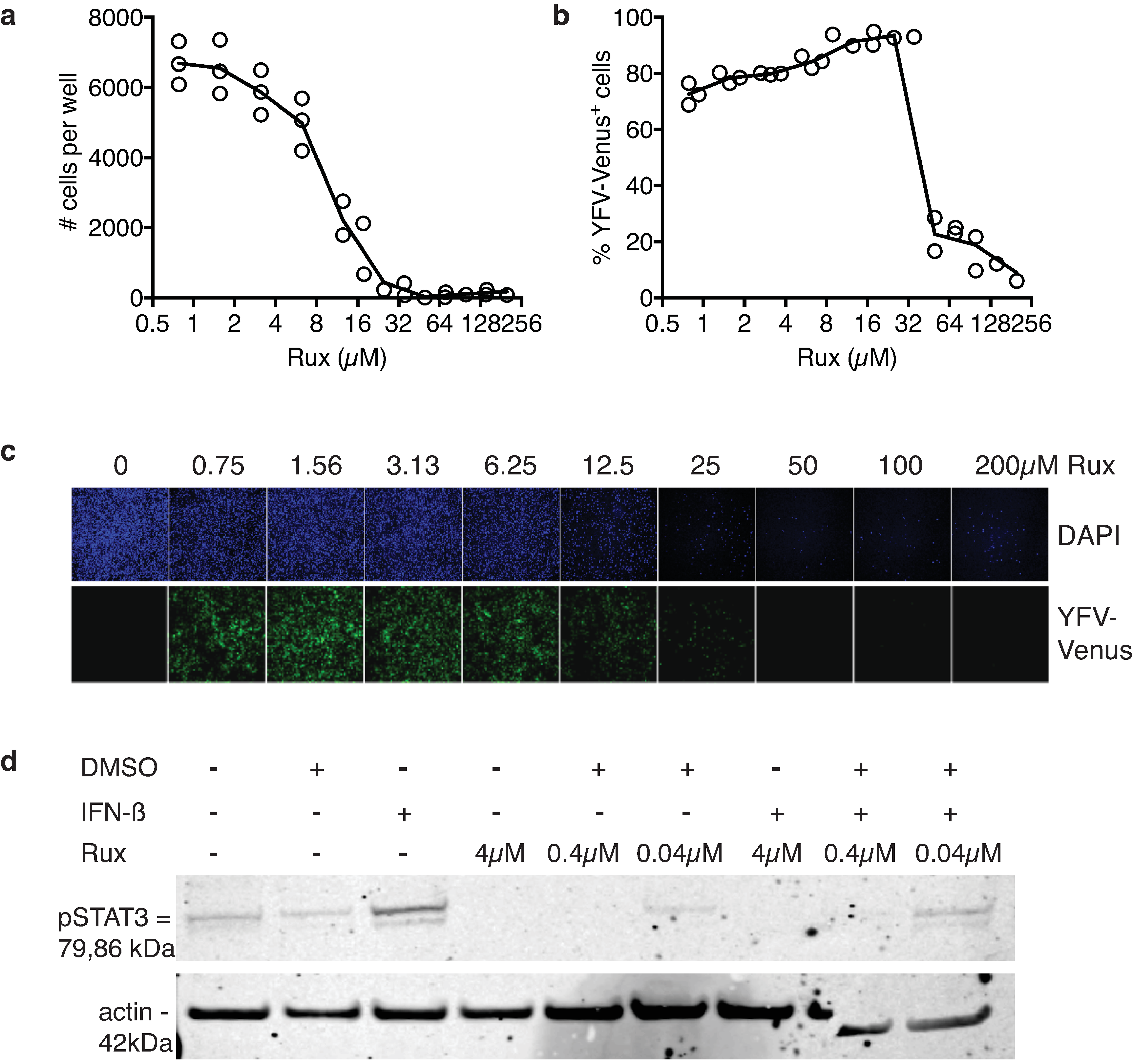

Supplement: S7 Fig — A549 cells were treated with indicated amounts of the pan-Jak inhibitor Ruxolitinib (Rux), or DMSO carrier control, and infected with YFV-Venus. Cells were imaged and cell numbers or YFV-Venus positive cells determined by microscopy. A. Cell count per well (DAPI-positive) 72 post Rux treatment. b. % YFV-Venus positive cells. c. Representative images of (a) and (b). d. Analysis of STAT3 phosphorylation as a readout of Jak activity. A549 cells were treated with 500 U/ml of interferon-beta, indicated amounts of Rux, or DMSO carrier control. At 48h post treatment, cells were harvested and analyzed by western blotting using anti-pSTAT3 antibody, or anti-actin antibody as loading control. (TIF) [file ppat.1007634.s007.tif]

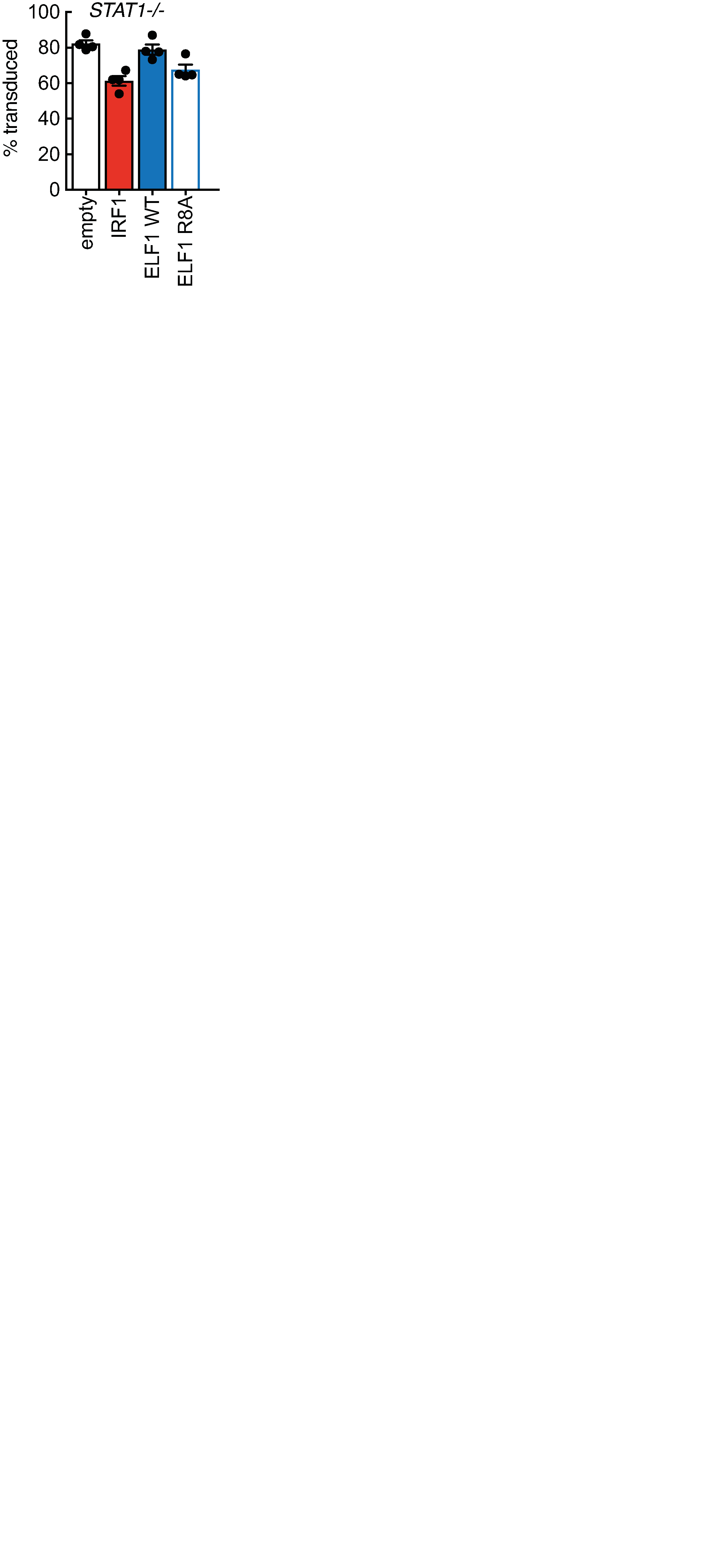

Supplement: S8 Fig — A549 STAT1-/- cells were transduced to express ELF1 or controls. 48 h post transduction, cells were challenged with a low MOI of influenza A/WSN/1933 virus. Mean +/- SEM of % RFP-positive (transduced) cells was determined by high content microscopy at 36 hpi (endpoint of experiment). (TIF) [file ppat.1007634.s008.tif]

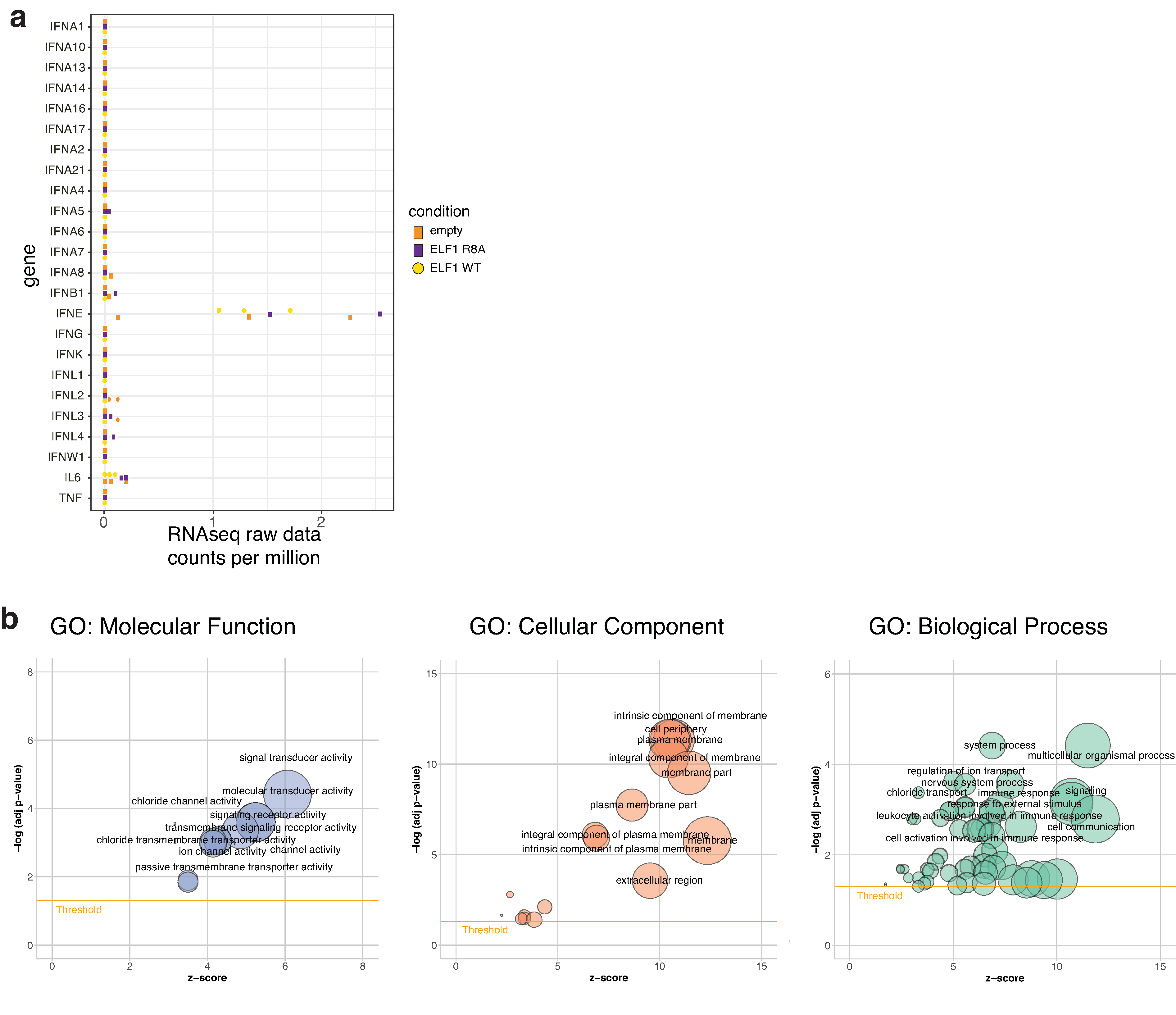

Supplement: S9 Fig — a. RNA-Seq read counts per million (not normalized) from Fig 6 (b) for type I, II and III interferons and additional select cytokines (Ensembl v75). b. “Bubble plot” depicting GO terms enriched in ELF1 differentially expressed genes. Each bubble represents a significant (GOSeq adjusted p-value < 0.05) GO term. y-axis indicates enrichment significance (-log10 adjusted p-value) and x-axis indicates gene expression fold-change score ([upregulated genes–downregulated genes]/ √number of genes]) for term member genes. Bubble size is proportional to the number of term member genes. GO categories (Biological Process, Cellular Component, Molecular Function) are presented as separate panels to facilitate visualization. Highly significant enriched GO terms (adjusted p-value < 10−3) are annotated. (TIF) [file ppat.1007634.s009.tif]

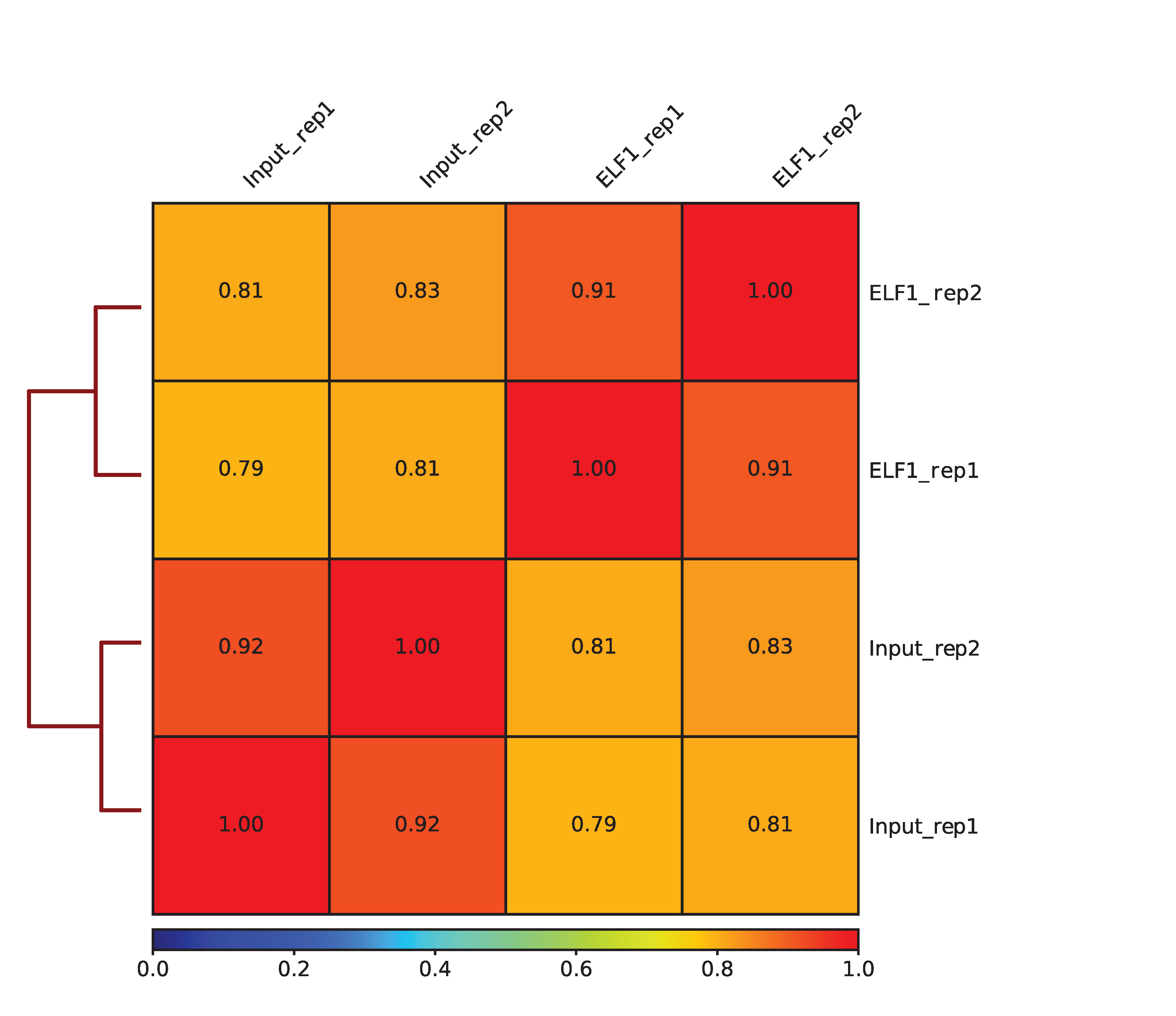

Supplement: S10 Fig — Pearson correlation of ELF1 ChIP and Input replicates. Read coverages were calculated for aligned reads from two ELF1 ChIP biological replicates and two Input control biological replicates with a bin size of 1kb and step size of 100 bp. Shown is a heatmap of pairwise Pearson correlation values of read coverages between the different samples. (TIF) [file ppat.1007634.s010.tif]
